# CAR-T lymphocyte-based cell therapies; mechanistic substantiation, applications and biosafety enhancement with suicide genes: new opportunities to melt side effects

**DOI:** 10.3389/fimmu.2024.1333150

**Published:** 2024-07-18

**Authors:** Paula Ercilla-Rodríguez, Marta Sánchez-Díez, Nicolás Alegría-Aravena, Josefa Quiroz-Troncoso, Clara E. Gavira-O'Neill, Raquel González-Martos, Carmen Ramírez-Castillejo

**Affiliations:** ^1^ ETSIAAB, Universidad Politécnica de Madrid, Madrid, Spain; ^2^ Laboratorio Cancer Stem Cell, HST group, Centro de Tecnología Biomédica, Universidad Politécnica de Madrid, Madrid, Spain; ^3^ Grupo de Biología y Producción de Cérvidos, Instituto de Desarrollo Regional, Universidad de Castilla-La Mancha, Albacete, Spain; ^4^ Asociación Española Contra el Cáncer (AECC)-Fundación Científica AECC, Albacete, Spain; ^5^ Sección de Oncología, Instituto de Investigación Sanitaria San Carlos, Madrid, Spain

**Keywords:** CAR-T, cell therapy, immune response, adoptive immunotherapy, gene therapy, T lymphocytes, oncology

## Abstract

Immunotherapy has made significant strides in cancer treatment with strategies like checkpoint blockade antibodies and adoptive T cell transfer. Chimeric antigen receptor T cells (CAR-T) have emerged as a promising approach to combine these strategies and overcome their limitations. This review explores CAR-T cells as a living drug for cancer treatment. CAR-T cells are genetically engineered immune cells designed to target and eliminate tumor cells by recognizing specific antigens. The study involves a comprehensive literature review on CAR-T cell technology, covering structure optimization, generations, manufacturing processes, and gene therapy strategies. It examines CAR-T therapy in haematologic cancers and solid tumors, highlighting challenges and proposing a suicide gene-based mechanism to enhance safety. The results show significant advancements in CAR-T technology, particularly in structure optimization and generation. The manufacturing process has improved for broader clinical application. However, a series of inherent challenges and side effects still need to be addressed. In conclusion, CAR-T cells hold great promise for cancer treatment, but ongoing research is crucial to improve efficacy and safety for oncology patients. The proposed suicide gene-based mechanism offers a potential solution to mitigate side effects including cytokine release syndrome (the most common toxic side effect of CAR-T therapy) and the associated neurotoxicity.

## 1 Introduction

Cancer is an umbrella term for a wide range of diseases characterized by cell heterogeneity. Tumor cell populations have distinctive characteristics: many of them show uncontrolled proliferation, little differentiation, and some of these cells have the ability to spread to adjacent as well as distant tissues or organs in a process called metastasis (1) with self-renewal capacity (2–4).

**Figure 1:**
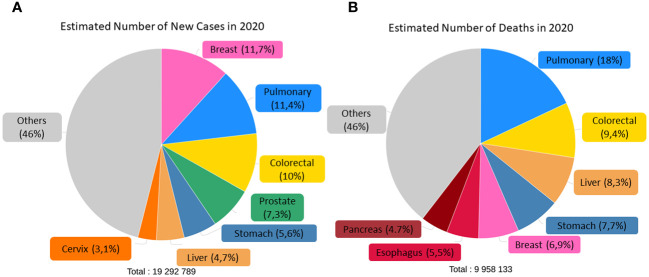
Estimated number of incidence **(A)** and deaths **(B)** of both sexes and ages in the world classified by cancer type. Adapted from: (6).

According to the World Health Organization (WHO), cancer is one of the leading causes of morbidity and mortality in the world, both in adults and children, second only to cardiovascular diseases. In 2020, there were an estimated 19.3 million new cases and 9.96 million deaths (5), and, consistent with the Global Cancer Observatory (GLOBOCAN) database, the incidence is expected to reach 28.4 million cases in 2040, an almost 50% increase compared to recent years. This is due to increased life expectancy and population levels, as well as massive exposure to various risk factors. Globally, the highest incidence rates are for breast (11.7%), lung (11.4%), colorectal (10%), prostate (7.3%) and stomach (5.6%) cancer, with the highest mortality rates for lung (18%), colorectal (9.4%), liver (8.3%), stomach (7.7%) and breast (6.9%) cancer, as shown in **Figure 1**.

Cancer is not caused by a single factor but is the result of the interaction of different risk factors, with 90-95% being caused by environmental factors and the remaining 5-10% having an inherited component. Smoking is the main risk factor and cause of lung cancer, also increasing the risk of other types of cancer. Other known risk factors are ageing, alcohol, obesity, ultraviolet radiation, and even different infectious agents such as the Human Papilloma Virus or the Human Immunodeficiency Virus, among others (7, 8). For this reason, it is considered a multifactorial disease capable of producing damage and genetic heterogeneity within the affected populations, greatly complicating the design of new therapies.

Research is currently focused on finding effective treatments that show a decrease in deaths, an increase in survival rate and progression-free time, as well as aiming to improve the response to ‘conventional’ treatments such as chemotherapy or radiotherapy. One of the most popular treatments in research currently is immunotherapy, based on the idea of increasing the immune system’s ability to fight disease, such as by use of chimeric antigen receptor-transduced T lymphocytes (CAR-T). The primary objective of this literature review is to describe CAR-T lymphocyte-based therapy and to this end, we will describe the main molecular basis and mechanisms of the immune response to tumors, summarize the different immunotherapies that led to the development of CAR-Ts, analyze the clinical status of CAR-Ts by detailing their role in cancer treatment, and assess the obstacles and future prospects of these treatments.

## 2 Relevant sections and discussion

### 2.1 Anti-tumor immunity

The immune system is comprised of a set of cells, tissues and organs that work together to activate an immune response, encompassing the mechanisms and processes for recognizing and defending against tumor cells in addition to invading agents. It is a highly coordinated and complex response affected by the immunological microenvironment and tumor microenvironment. The number of newly described targets increases frequently, (9, 10) particularly consisting of immune and non-immune cell components (10, 11), demonstrating the essential role of the tumor immune microenvironment in understanding tumor biology (12) which can be summarized in the three following scenarios.

#### 2.1.1 Anti-tumor immunity and immunogenic death

**Figure 2:**
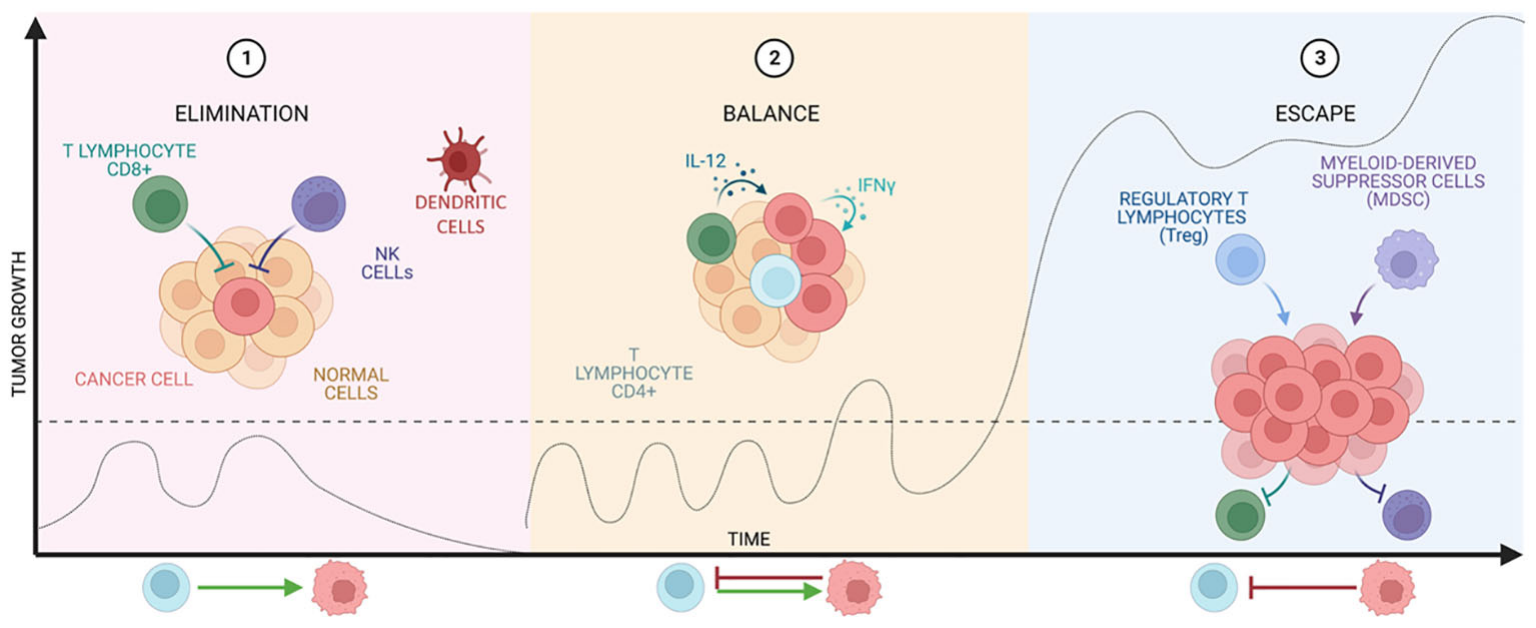
Stages of cancer immunoediting. The graph shows tumor growth at the different stages of immunoediting developed by Thomas and Burnet.

The immune system (IS) plays a central role in the elimination of tumor cells. This role was proposed by Paul Ehrlich and later studied in depth by Thomas and Burnet, who proposed the term “immunosurveillance”, referring to the immune system’s ability to detect and eliminate tumor cells, preventing the development of disease (13). Nowadays, this concept has been further extended to immunoediting, which consists of three stages: elimination, equilibrium, and escape, seen in **Figure 2**.

The elimination phase represents the classic idea of immunosurveillance proposed by Burnet. After transformation has occurred and intrinsic non-immunological tumor suppressive mechanisms have failed (14), the innate and adaptive immune systems are co-activated to destroy the tumor (15). This combination is able to recognize tumor antigens and to develop an appropriate effector response on the transformed cells that express them (16, 17).

However, if the tumor is not eradicated, the equilibrium phase begins, in which growth is immunologically restricted, but not completely eliminated. The duration of this phase varies from patient to patient as it is influenced by the structure and the established tumor suppressive environment (TME). Finally, this state of equilibrium can lead to tumor escape (18–20), where tumor cells recruit IS cells to create an immunosuppressive TME and these cells in turn induce tumor cells to express immune checkpoint molecules (15) and even inhibitors (21, 22).

#### 2.1.2 Cycle of anti-tumor immunity

**Figure 3:**
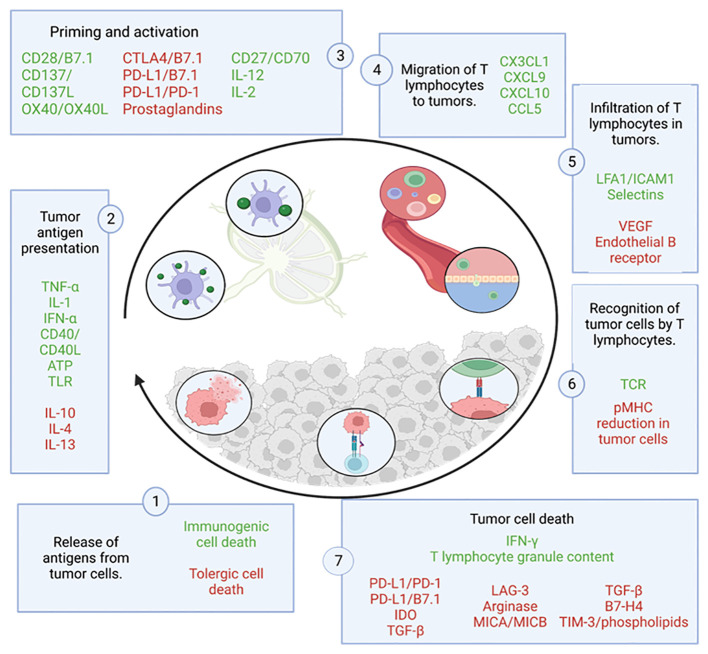
Cycle of the anti-tumor immune response. This figure shows the different steps that make up the tumor immunity cycle together with the factors that stimulate (in green) or inhibit (in red) tumor cell death. Adapted from: (23).

Focusing on the cycle of the anti-tumor immune response (**Figure 3**), it begins with the release and capture of neoantigens, generated during oncogenesis, by antigen-presenting cells (APCs), such as dendritic cells (DCs). These cells process the antigens and undergo a maturation process, which involves an increase in the expression of various molecules, such as the chemokine receptor CCR7 (24–26)and the migration of DCs to the lymph nodes where they can interact with T cells (24), driving complex collective migration patterns, allowing DCs to create or enhance chemotactic gradients (24). For an anti-tumor T response to be generated, this process needs to be accompanied by signals that prevent peripheral tolerance to tumor antigens, such as high expression of major histocompatibility complex (MHC) molecules and co-stimulatory molecules (such as CD80, IL1, IFN-α) (27). This allows antigenic presentation via MHC I to effector T cells (CD8+ T cells) or MHC II to helper T cells (CD4+ T cells) (27), which triggers priming and activation of effector T responses directed against these antigens. At this point, the balance between regulatory T cells (Treg) and effector T cells determines the nature of the immune response and its outcome.

CD8+ T cells are a cell population that can differentiate into several subgroups depending on the environmental stimuli they receive, which affects their function. The most studied and relevant subgroup is the subpopulation of cytotoxic CD8 T cells, also known as Tc1 (28). This subgroup is able to recognize and bind by interaction to its T-cell receptor (TCR) with the cognate antigen bound to MHC I, leading to the elimination of tumor cells by: synaptic exocytosis of granules composed of granzymes and perforins known as the “kiss of death”, or indirectly by secretion of cytokines such as tumor necrosis factor (TNF) or interferon gamma (IFNγ) (29). The death of these cells releases more tumor antigens, which positively feed back into the anti-tumor response in successive cycles (23).

#### 2.1.3 Evasion of the immune response by tumors

It is now clear that the immune system can defend itself and attack tumor cells. However, the high prevalence, relapses and deaths indicate that this immune response is often ineffective. It is therefore recognized that many tumors have the ability to evade the host immune system. They exit the elimination and balance phase and enter the so-called escape phase. Consequently, one of the major goals of immunology is to understand these escape mechanisms in order to neutralize them and thus stop tumor growth. Some processes and molecules involved in this evasion are being brought to light, molecules actually previously linked to tumor progression such as ALDH2 and which have now been linked to tumor evasion of the immune response (30).

One such mechanism employed by the tumors is the inhibition of immune checkpoints. These checkpoints are molecules that normally regulate the immune response and prevent autoimmunity. However, tumors exploit self-expression of these molecules, such as CTLA-4 (cytotoxic T-lymphocyte-associated protein) and PD-1 (programmed cell death protein 1), to inhibit the response of tumor-infiltrating T lymphocytes (TILs), as they are two major negative regulators of the anti-tumor response. CTLA-4 is an inhibitory protein expressed on the surface of activated T cells. When activated, CTLA-4 translocates to the cell surface and binds to B7 molecules (CD80/CD86) on APCs. This interaction inhibits the activation and uncontrolled proliferation of T cells, thus limiting the immune response. On the other hand, PD-1 is an inhibitory protein expressed on the surface of several types of immune cells, including T lymphocytes, and can bind to its ligands, PD-L1 and PD-L2, inhibiting T-lymphocyte activation and effector function. This prevents an exaggerated immune response and autoimmune damage. However, some tumor cells may have the ability to express both CTLA-4 and PD-L1 (31, 32); inhibiting the antitumor response, and blocking this expression is the basis for the immunotherapy awarded in 2018 with the Nobel Prize in Physiology or Medicine to James P. Allison and Tasuku Honjo.

**Figure 4:**
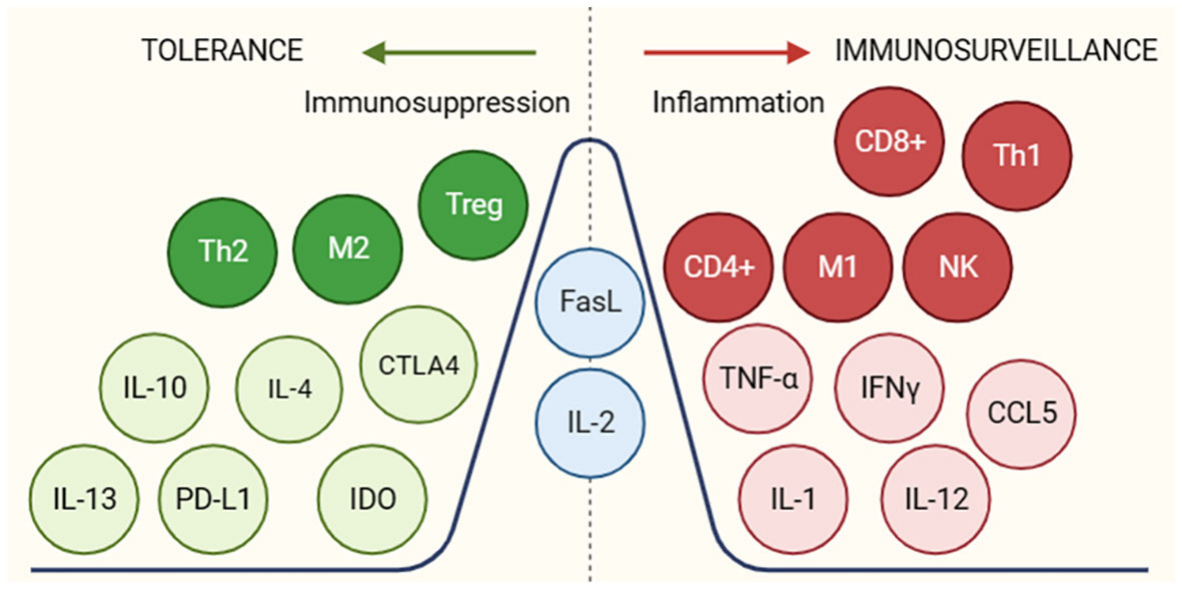
Molecules and cellular subgroups involved in the induction of an immunosuppressive environment or active immune response. The different molecules involved in promoting a tol-erant (green) or immunogenic (red) environment are shown. In blue are molecules that may be-long to both groups depending on the environment in which they are found. For the function of the respective molecules, see **Supplementary Table S2**.

Another tumor escape mechanism is the secretion of molecules to induce inflammatory and immunosuppressive responses in the tumor microenvironment (**Figure 4**) promoting tumor escape and progression. Some of these molecules are transforming growth factor beta (TGF-B), interleukin-10 (IL-10), interleukin-4 (IL-4) and the onco-immunogenic molecule CD155 overexpressed in tumor microenvironments (TME), which all play multiple immunosuppressive roles. CD155+ tumor-associated macrophages showed an M2 phenotype and higher expression of interleukin (IL)-10 and transforming growth factor (TGF)-β (33). These cytokines inhibit the proliferation and effector functions of lymphocytes (B and T) as well as macrophages, limiting their ability to recognize and eliminate tumor cells. In addition, they activate Treg, thus contributing to the maintenance of a tolerogenic profile. These molecules also promote the differentiation and accumulation of myeloid-derived suppressor cells (MDSCs) in bone marrow, lymphoid tissues and in the tumor microenvironment. These suppressor cells inhibit innate and T-lymphocyte mediated anti-tumor immune responses by secreting immunosuppressive cytokines that possess the ability to modulate the tumor microenvironment (by decreasing the production of pro-inflammatory cytokines) creating a favourable environment for tumor growth and evasion (34).

Finally, the loss of tumor antigen expression is also a well-known escape mechanism (35–39). Due to the high mitotic rate of tumor cells and their genetic instability, it is common to find mutations in genes encoding these antigens. In some cases, these antigens are not critical for the survival or maintenance of the phenotype of the transformed cell and will thus go unnoticed by the immune system. In addition, a decrease in MHC-I expression is observed, preventing recognition of tumor cells by CD8+ T cells. This may be due to a reduction in the synthesis of the MHC-I complex or alterations in components involved in antigen processing such as TAP1, TAP2 or proteasome subunits (40).

In order for immunogenic cell death (ICD) of tumor cells to occur, it is important to have a number of factors (**Table 1**) such as: antigenicity, adjuvanticity (release of cell damage-related molecular patterns (DAMPs) due to stress or cell damage) (42) and a permissive microenvironment (41, 43, 44).

**Table 1:** Different possible effects generated as a function of factors that establish immunogenic cell death.

MMUNOGENIC CELL DEATH				
-	+	+	+	+
ANTIGENICITY				
-	-	+	+	+
ADJUVANTICITY				
-	+	-	+	+
TUMOR MICROENVIRONMENT				
+/-	+/-	+/-	-	+
EFFECT				
NO RESPONSE	INFLAMMATION	TOLERANCE	PRIMING	EXECUTION

Table adapted from: (41).

Regarding antigenicity, in the absence of antigens not subject to central or peripheral tolerance, ICD can only trigger inflammatory responses without activating adaptive immunity. However, when antigenicity is present and sufficient adjuvanticity is present, ICD can activate the adaptive response. Otherwise, a state of tolerance would be induced. In the case of tumors, the high rate of mutations in the coding regions of the genome (non-synonymous and frameshift point mutations) can lead to exposure of tumor neoantigens, increasing antigenicity. In addition, some cancer cell autoantigens can also induce anti-tumor immunity (42).

On the other hand, adjuvanticity can be induced by cancer cells by the release of DAMPs, such as interferon (IFN), adenosine triphosphate (45) and calreticulin along with related cytokines. These molecules play an important role in the activation of adaptive immunity through the involvement of pattern recognition receptors (PRRs). However, it is important to note that DAMPs cannot initiate an immune response without increased antigenicity in dying cells (42, 46).

Finally, the tumor microenvironment determines whether T cells, activated by ICD-responsive dendritic cells, can access the tumor bed to carry out an effector response and establish memory. Factors such as the extracellular matrix (ECM), the macrophage polarization towards a pro-inflammatory (M1) or anti-inflammatory (M2) phenotype and the lymph nodes draining the tumor also influence this process (42).

Thus, all these tumor escape mechanisms allow cancer cells to evade immune surveillance and develop strategies to survive and grow without being attacked by the immune system.

### 2.2 Cancer immunotherapy

**Figure 5:**
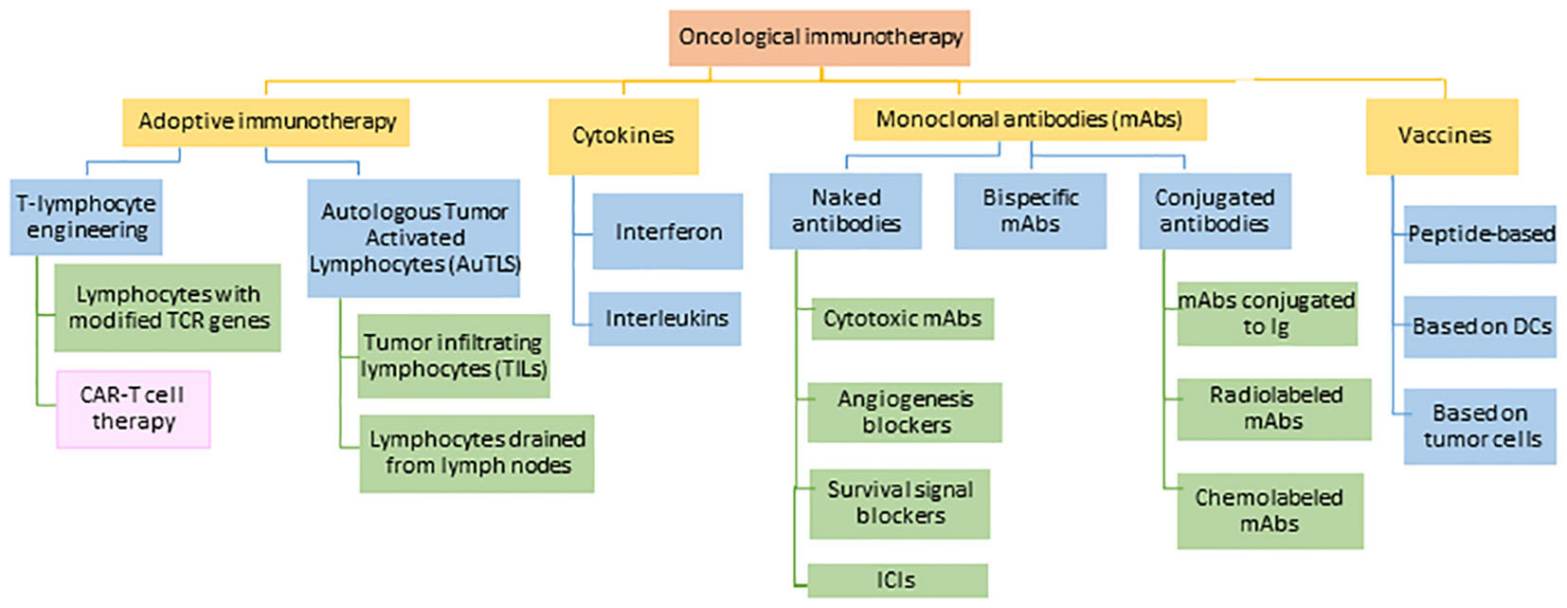
Current approaches in the field of cancer immunotherapy. TCR: T-lymphocyte receptor, CAR-T: chimeric antigen receptor for T-lymphocytes, DCs: dendritic cells, Ig: immunoglobulin, ICIs: immune checkpoint inhibitors. Adapted from: (55).

The term ‘cancer immunotherapy’ refers to any type of treatment based on stimulating and using the patient’s own immune system to attack the cancer or block cancer escape routes (47). Looking back, the first attempts to modulate the immune system to treat cancer are attributed to the German physicians Fehleisen (48) and Busch (49), who observed significant tumor regression after infection (50). Later, William Bradley Coley (51), known as the father of immunotherapy (52), attempted to harness the immune response to treat sarcomas in 1891 (53, 54). However, his achievements went unnoticed until half a century later when several fundamental discoveries in the field of immunology related to T cell existence and function led to an expansion in the study of immunotherapy, opening a new era in cancer treatments. Thanks to these therapies, long-lasting and successful responses have been observed in patients with advanced cancers (52). One of the most important and most advanced treatments in the fight against cancer is chimeric antigen receptor therapy or CAR-T, which involves modifying the patients’ own T cells. The mainstays of this therapy are immunotherapies with monoclonal antibodies (mAbs) and adoptive T-cell transfer (ACT) (**Figure 5**).

#### 2.2.1 Modulatory monoclonal antibodies

Immunomodulatory mAbs are proteins designed *in vitro* to mimic the function of natural antibodies. The characterization of tumor-associated or tumor-specific antigens allows us to generate specific mAbs against them, which can later prevent the antigen from binding to its receptor on the cell surface or mark the antigen for destruction by complement-mediated cytotoxicity (CDC) or antibody-dependent cytotoxicity (ADCC), among other uses. However, a major obstacle is the identification of cancer cell antigens that can be targeted without damaging healthy tissues (52).

The first mAb approved by the Food and Drug Administration (FDA) in 1997 to treat cancer, specifically Non-Hodgkin’s lymphoma, was Rituxinab (56) which specifically binds to the CD20+ molecule resulting in the depletion of CD20+ B cells (57).

Today, the most widely used monoclonal antibody therapy is the modulation of immune checkpoint inhibitors (ICIs), particularly CTL-4, PD-1 and their ligands (PD-L1, PD-L2) (58). Following this strategy, the FDA has approved three different categories of ICIs. These are PD- 1 inhibitors (Nivolumab, Pembrolizumab and Cemiplimab), PDL-1 inhibitors (Atezolimunab, Avelumab and Durvalumab) and CTLA-4 inhibitors (Ipilimunab) (59).

#### 2.2.2 Adoptive T-cell transfer

ACT is a highly personalized therapy that aims to induce a potent anti-tumor response, directly or indirectly, through the infusion of immunologically active T cells after being manipulated *ex vivo* and *in vitro* (47).

**Figure 6:**
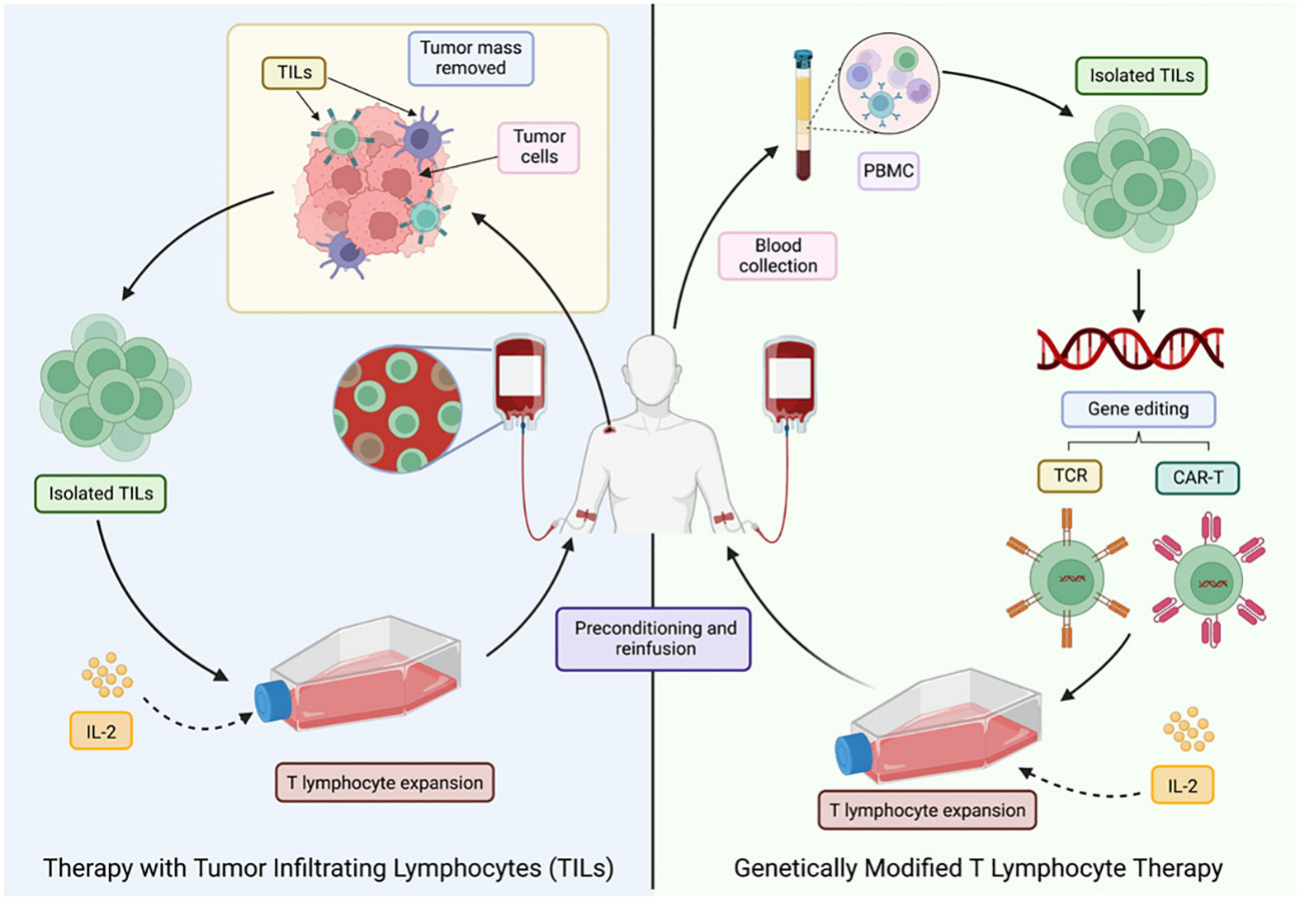
Different ACT strategies for cancer treatment. LT, T lymphocytes; TILs, tumor-infiltrating T lymphocytes; PMBC, peripheral blood mononuclear cells. Adapted from: (47).

There are different sources for obtaining these cells: *in vitro* differentiated stem cells, compatible donors (allogeneic transplantation) and the patients’ own cells (autologous transplantation) (60). The latter option is the preferred route as it avoids rejection or graft-versus-host disease. Autologous transplantation can be divided into two main strategies as shown in **Figure 6**.

The first approach was developed in 1980, after the technique for the generation of human lymphoid cells with the ability to lyse tumor cells while sparing healthy cells was first described. This was achieved by incubating peripheral blood lymphocytes with Interleukin-2 (IL-2), termed lymphokine-activated killer cells (LAKs) (61). These activated cells were then re-infused into the patient, who also received a large dose of IL-2. However, despite the positive results, it was noted that a large number of patients experienced toxicities associated (62) with the high doses of IL-2 required to achieve an effective anti-tumor response (47).

In 1982, therapy with TILs, a heterogeneous group of lymphocytes (T and NK) (47), with the natural ability to recognize and attack cancer cells, was pioneered, making them the most effective effector cells of the immune system in the fight against cancer (63). TILs recognize tumor-associated antigens (TAAs) through the endogenous TCR (47), but the main drawback is the limited number of lymphocytes available, as well as the high costs, time (6-8 weeks) and difficulty to standardize their production (64).

### 2.3 What is CAR-T?

Building on the types of immunotherapies described above, chimeric antigen receptors (CARs) have emerged as a promising technology in cancer treatment. These synthetic proteins are designed to be expressed on the surface of cytotoxic cells of the immune system (IECS), in order to enhance the recognition and killing of specific cells, including tumor cells (65).

An example of the use of chimeric antigen receptors are CAR-T lymphocytes. These are T-lymphocytes that are genetically modified to express the synthetic receptor on their cell surface. This receptor gives them the ability to specifically recognize and attack cancer cells by targeting specific tumor antigens (66). This process combines the cytotoxic properties of the T-lymphocyte with the specificity provided by a high-affinity recognition domain, usually derived from a monoclonal antibody (mAb) (67).

#### 2.3.1 Structure of chimeric antigen receptors

**Figure 7:**
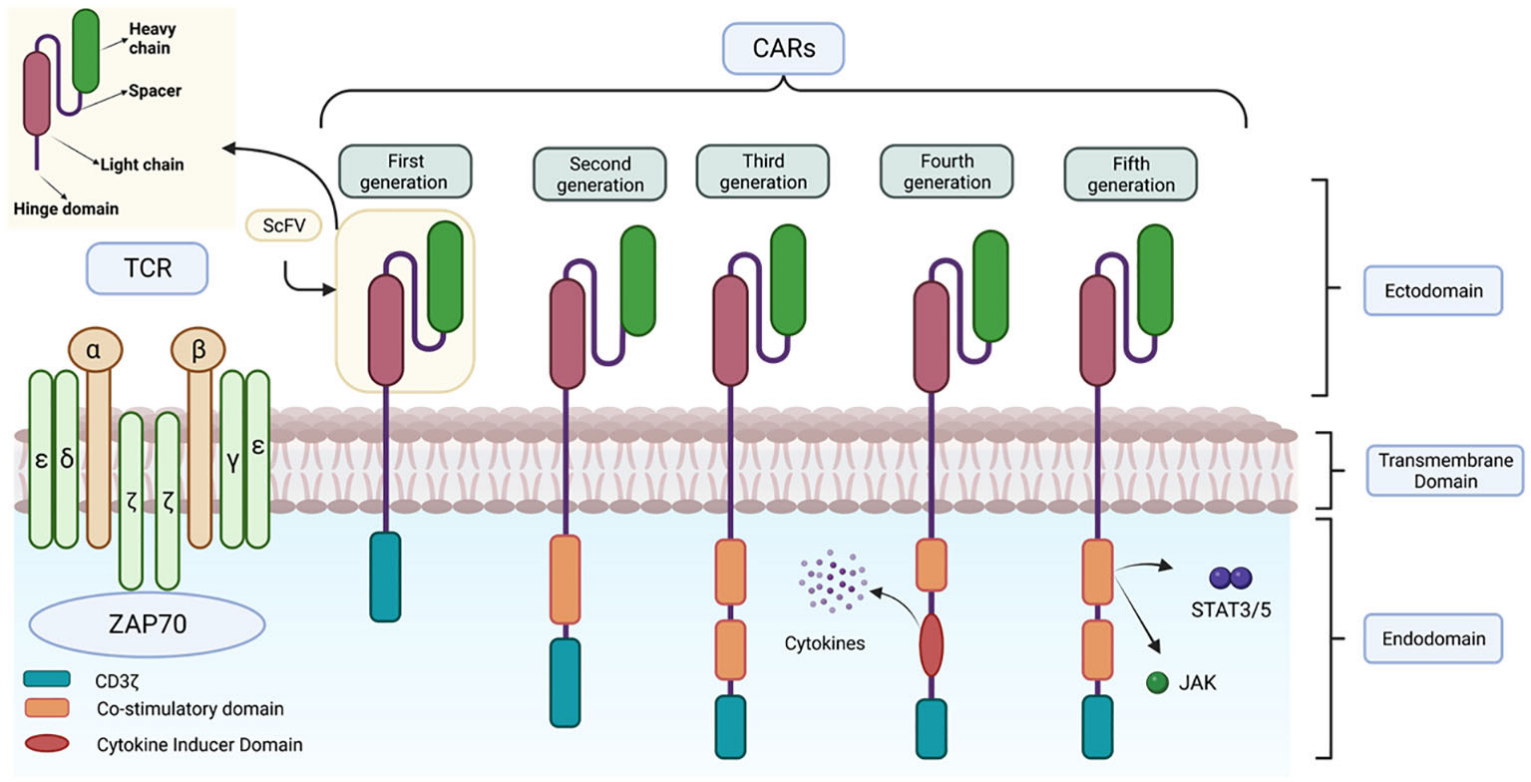
Representation of the structure of the different CAR generations. This figure shows the main differences in the structure of the different generations of CARs together with the structure of the TCR.

CARs are composed of four distinct domains (**Figure 7**): the ectodomain or extracellular domain, the hinge domain, the transmembrane domain and the endodomain or intracellular signalling domain (68). Each of these domains plays an important role in the function of the receptor and therefore the precise composition of each domain is crucial for its proper functioning (69–71).

##### 2.3.1.1 Ectodomain

The extracellular domain or ectodomain (ECD) plays a key role in target antigen specificity as it contains the antibody responsible for antigen recognition.

It is formed from the single-stranded variable fragment (scFv) consisting of the variable regions of the heavy and light chains of an immunoglobulin, fused by a spacer peptide of approximately 15 residues in length (72). This domain is part of a synthetic antibody with a higher affinity for pMHC (MHC peptide) compared to the TCR. The scFv domain (73) allows recognition of specific cell surface antigens, including proteins, glycoproteins, carbohydrates, among others (74). This extends the reach of CAR-T beyond TCR-restricted MHC-peptide targets as CAR-T cells have the ability to recognize tumor cell surface antigens without the MHC complex, allowing them to recognize a wide variety of targets on tumor cells without being affected by the loss of MHC expression. For example, in solid tumors the antigen of the ectodomain could recognize IgC and IgV domains of the B7-H3 protein family (75). It should be noted that although after binding to the antigen the scFv fragment has been shown to trigger an activation signal through the endodomain and activation of different signalling cascades, the exact mechanism by which this occurs is still unknown.

In recent years, new alternatives to the use of the scFv domain as the main target interaction domain have emerged. Among these alternatives are nanobodies (also called single domain antibodies or VHH), derived from the variable domain of naturally occurring heavy chain only antibodies (HcAbs) first described in camelids. They present several advantages over traditional scFvs (76) such as lower immunogenicity due to the absence of synthetic binding peptides and the elimination of the murine origin of the scFv (71). In addition, they maintain similar binding capacity and specificity to traditional scFvs even in the absence of the variable light chain and constant domains (77). Nanobodies also prevent self-aggregation of the scFv domain, which prevents premature antigen-independent depletion of CAR-Ts, which can be caused by exposure of hydrophobic residues on both variable chains (78).

Another alternative consists in the use of dual chains, whereby the antibody-binding domain is used in its natural heterodimer form, consisting of the simultaneous expression of the light and heavy chain of an immunoglobulin, linked by endogenous disulfide bonds to its constant region. This structure enhances receptor stability and prevents antigen-independent clustering in the same way as nanobodies (79).

In addition to these, native receptors and ligand-based receptors have been developed as alternatives to this domain. These alternatives reduce immunogenicity due to their smaller size, decreasing the risk of triggering an adverse immune response and leading to increased persistence of CAR-Ts (65).

##### 2.3.1.2 Hinges and transmembrane domain

The ectodomain connects to the transmembrane domain via a spacer known as a hinge (71). This spacer is usually derived from the constant fraction (Fc) of the immunoglobulin subfamily IgG (mainly IgG1 or IgG4), IgD or the CD8 domain (80). Although little research has been done on this component, it plays an important role in CAR-T activation, as the spacer design can modulate synaptic cleavage distances, provide flexibility (removing steric hindrance) and promote dimerization (increasing the strength of the activation signal), as well as reduce non-specific innate immune responses (81). It has also been observed that some IgG-derived peptides can interact with Fcγ receptors (FcγR), leading to CAR-T cell depletion and decreased persistence *in vivo*. Therefore, research is underway to optimize this domain through point mutations that prevent these interactions (82).

On the other hand, the transmembrane domain is located between the hinge domain and the endodomain and acts as an anchor point for the transduction of recognition signals of ligands to the intracellular domain. This domain is usually derived from CD3-ζ, CD4, CD8, ICOS or CD28 molecules. Although transmembrane domains used to be considered structural ligands of little interest, they are now known to have the ability to influence the effector function of CAR-Ts (69). For example, CARs containing the CD28 transmembrane domain tend to be more stable than those with CD3-ζ, but the latter can dimerize and facilitate activation. In addition, the CD8 domain releases less IFNγ and TNF and is less susceptible to activation- induced cell death (AICD) than those derived from CD28 (83).

##### 2.3.1.3 Intracellular domain or endodomain

The intracellular domain or endodomain consists of the zeta chain of the TCR immunoglobulin receptor (TCR CD3ζ) (72) or, alternatively, the intracellular signalling domain of another protein containing an immune receptor tyrosine-based activation motif (ITAM); which is sufficient to induce antigen-dependent T cell activation (54). However, as activation of these genetically modified cells is dependent on exogenous IL-2, *in vivo* studies have shown reduced expansion, stability and anti-tumor activity of these CAR-T cells, as they lack interaction with co-stimulatory receptors. To address these limitations, subsequent generations of CARs have been developed (**Figure 7**) (84).

In 2nd generation CARs, a single costimulatory domain, such as CD28 or 4-1BB, is added between the transmembrane domain and CD3ζ (65). The CD28 domain activates the signalling pathway via the enzyme phosphatidyl-inositol 3-kinase (PI3K), which induces massive T cell proliferation and the production of proinflammatory cytokines such as IL-2, enhancing the persistence and duration of CAR-T cells *in vivo* and *in vitro*. On the other hand, 4-1BB (CD137), activates nuclear factor NK-KB, mitogen-activated protein kinase (MAPK/ERK) signalling pathways (85, 86). These are currently used in clinically approved therapies such as Kymriah^®^ and Yescarta^®^.

Third-generation CARs are characterized by the simultaneous incorporation of two costimulatory domains, which can be a combination of CD28, ICOS (promotes Th17 polarization), OX40 and 4-1BB. This strategy allows for greater therapeutic efficacy compared to previous generations, as greater persistence, less differentiation and depletion are observed (87). The combined use of these costimulatory domains has been shown to be highly effective in killing cancer cells (81, 87).

TRUCKs (CAR-redirected T cells for universal cytokine killing) represent the fourth generation. These lymphocytes are derived from the third generation and are used as vehicles to produce and release specific pro-inflammatory cytokines to overcome the suppression generated by the tumor microenvironment, recruit other immune system cells and activate the anti-tumor response (88). These cytokines are: IL-12, which enhances T-cell viability, recruits and activates other IS cells to improve potency and safety (42), as well as IL-15, IL-18, CCL19 and IL-7. This release can be constitutive or induced, e.g. by nuclear factor T-lymphocyte activating factor (NFAT) when the T cell is activated by CAR in the target tissue (55, 89).

The fifth generation CAR includes a truncated beta-chain domain of the cytoplasmic IL-2 receptor with a binding site for the transcription factor STAT3. Thus, when the lymphocyte is activated through the CAR, this receptor simultaneously activates the TCR, via CD3ζ, CD28 and the JAK-STAT3/5 signalling pathway, using the three intrinsic physiological activation pathways of T cells. As the most recent, its efficacy and safety is still under investigation (90).

**Figure 8:**
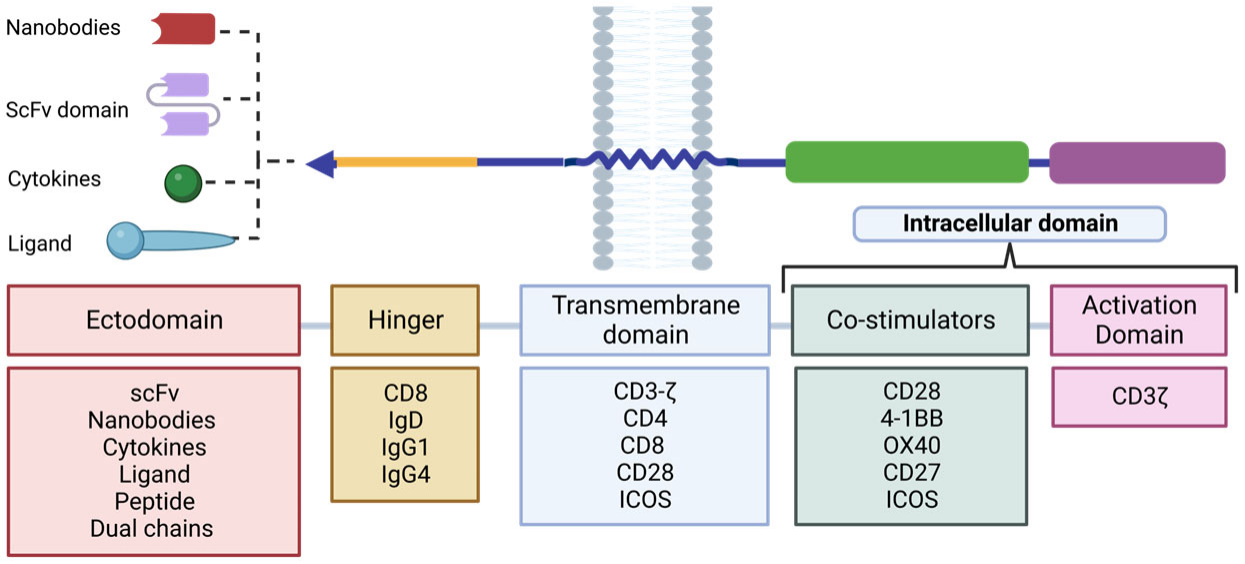
Design plan of the different RACs. The different components used for each domain that make up the RAC are shown. Adapted from: (83).

In second and subsequent generation CAR designs, the order of the costimulatory domains has been found to influence the effector function of CAR-Ts. For example, a trial to study the functional differences between CAR CD28-CD3ζ-CD28 and CD3ζ-CD28 CARs found that the former conformation secreted higher levels of IL-2 and exhibited sustained activity over time. Currently, the optimal combination of the different domains to achieve the highest CAR-T efficacy against the target antigen remains to be determined. Personalization of these domains (**Figure 8**) may be a key strategy to optimize the function of these cells and effectively direct them against the tumor depending on the target antigen of the desired outcome. This can lead to a more personalized and individualized therapy (81).

#### 2.3.2 Generation and manufacture of large-scale clinical-grade CAR-T lymphocytes

To achieve successful CAR-T therapy, it is crucial to ensure adequate transduction and expansion of the patient’s cells. This is especially important in patients who have received previous treatments, such as chemotherapy, which may compromise their immune system and hinder the expansion of CAR-T needed for therapy (91).

**Figure 9:**
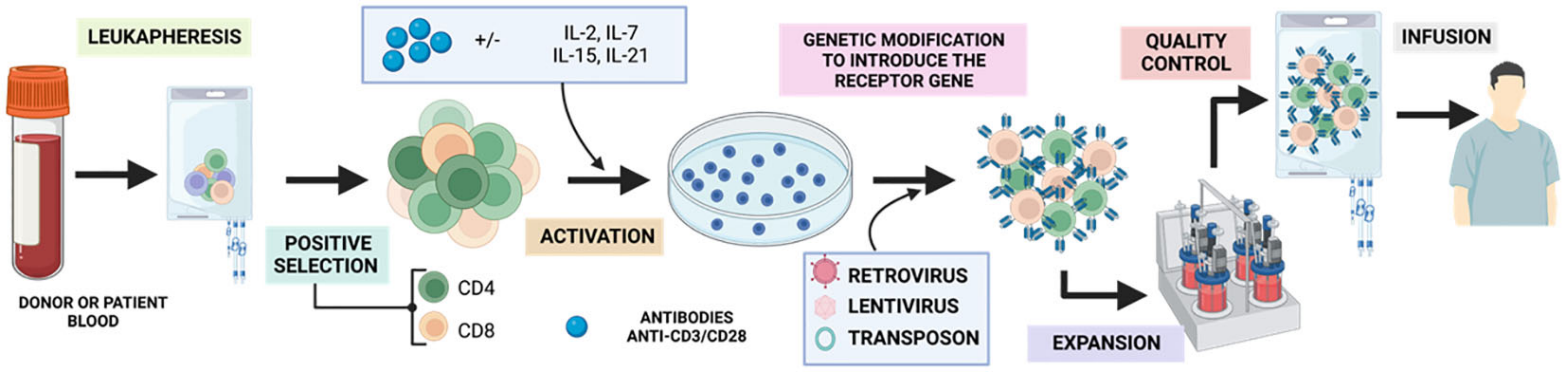
Generation of GMP-grade CAR-T. Adapted from: (92).

The process (**Figure 9**) begins with a peripheral blood leukapheresis, which involves the separation of leukocytes from the rest of the blood components. This is followed by positive selection of T cells using magnetic microbeads coated with anti-CD4 and anti-CD8 antibodies, which eliminates inhibitory and contaminating cells from the therapy (91). Subsequently, they are activated *in vitro* in a sustained manner for 48h with humanized anti-CD3/CD28 antibodies conjugated to a colloidal polymeric nanomatrix (93). This process takes place in the presence of a cytokine cocktail, which may vary depending on the study, and can include IL-2 or IL-7/IL-15 (94).

Once activated, the obtained T cells are modified by a transduction strategy that generally involves the use of retroviral or lentiviral vectors, or the transposon/transposase system (95) and expanded in a closed culture system, such as culture bags (35%), T-flasks (22%) or bioreactors (43%) between 7-11 days. Over the years, there has been an evolution from manual static cultures to using closed, automated bioreactors such as the ClinicMACS Prodigy (91). During expansion, a mixed CAR+ and CAR- product is obtained, with a variable proportion of CD4 and CD8 and a more effector, undifferentiated or memory phenotype depending on the starting material. Finally, the relevant control analyses such as immunological, microbiological and characterization analyses are performed to classify the product as suitable for clinical use under good manufacturing practices (95) and once accepted, the product will be injected into the patient.

To achieve successful CAR-T therapy, it is crucial to ensure adequate transduction and expansion of the patient’s cells. This is especially important in patients who have received previous treatments, such as chemotherapy, which may compromise their immune system and hinder the expansion of CAR-T needed for therapy (91).

During this production period, the patient undergoes lymphodepletion with chemotherapy (mostly cyclophosphamide/fludarabine). This increases the likelihood of a longer-lasting response as it favours the *in vivo* expansion of the administered CAR-Ts, as well as eliminating Treg that can compromise therapy (96).

##### 2.3.2.1 Target antigen determination and selection

Currently, one of the main challenges for the development of CAR-T therapies is the identification of appropriate and specific target antigens for each type of cancer, as the selection of these antigens has a significant impact on the specificity, efficacy and toxicity of the treatment, and thus its’ success (97, 98). The target antigen must meet certain requirements: be absent in healthy tissue or, if found, tolerate the cytotoxic activity of CAR-Ts. Two types of antigens can consequently be described: tumor-associated antigens (TAAs) and tumor-specific antigens (TSAs).

TAAs are molecules that are mimetically and aberrantly expressed on cancer cells, usually at much higher levels than on normal cells. In addition, many of these antigens are immunogenic, making them potential targets of the immune systems. These antigens are easy to identify and shared by many patients but targeting them poses a greater risk of serious effects due to their low specificity, which can result in “off-tumor on-target” toxicity. For example, HER2-targeted CAR-T cells can cause respiratory distress and cardiac arrest due to expression of this antigen on normal cells (99). **Supplementary Table S1** lists the main antigens used as targets, as well as the neoplasms and healthy tissues in which they are also present and, which are therefore, susceptible to the cytotoxic action of CAR-Ts. On the other hand, TAAs can also be found in extracellular vesicles originating from tumors and confounding CAR-Ts (100).

In contrast, TSAs are dysfunctional peptides derived from non-synonymous somatic mutations, such as single nucleotide variations (SNPs), deletions and insertions, among others. They are only expressed in tumor cells, which reduces the risk of off-tumor effects (98). Although TSAs are the ideal target for most immunotherapies, most CAR-Ts are directed towards TAAs, due to the low frequency of these specific mutations or TSAs. In addition, identifying these mutations at the transcriptomic or proteomic level and generating patient-specific CAR-Ts is costly (101).

On the other hand, the target antigen must be expressed on the cell surface. But, unlike natural T cells, CAR-Ts have the ability to recognize antigens without the need for them to be presented by the MHC, which broadens the spectrum of possible targets of natural T cells, enhancing the potential for therapy.

The antigen must be essential for the survival or growth of tumor cells, otherwise immunoediting could favour the proliferation of tumor clones lacking the target antigen, resulting in tumor escape.

Therefore, the ideal would be to characterize a tumor-specific antigen that is not present in any other healthy tissue and is vital for tumor survival, as well as being expressed on the cell surface. Although this may be a limitation, it should be noted that CARs can recognize different structures including proteins, carbohydrates and glycolipids, among others, increasing the set of available antigens (102).

Tian et al. (103) describe the use of bicistronic CAR-T cells that target multiple antigens expressed in neuroblastoma to overcome antigenic heterogeneity. This approach may lead to breakthroughs in the implementation of CAR-T cells for the treatment of solid tumors (104).

##### 2.3.2.2 Choice of vector

Lymphocyte modification requires effective and safe genetic engineering tools that are capable of introducing the genetic material of interest into the lymphocyte itself either *ex vivo* or *in vivo*. To introduce the gene that will encode the CAR receptor into the T lymphocyte and induce its expression on the cell membrane, there are different strategies, which are described below.

###### 2.3.2.2.1 Virals

Viral vectors use the natural ability of viruses to introduce genetic material into the cells they infect. These viruses are modified so that instead of their genetic material, they introduce the gene of interest by eliminating the virulence genes.

The most widely used are retroviral vectors [95% of all products manufactured (94)]. These vectors require the formation of viral particles containing the transfer plasmid encoding the transgene flanked by long terminal repeats (LTR) and the encapsidation signal Ψ (psi) (105). Among these vectors, lentiviral vectors derived from human immunodeficiency virus and gammaretroviral vectors, derived from Moloney murine leukaemia virus or murine cell virus, stand out (106).

The generation of the retroviral viral particle involves the introduction of basic genes required for survival and function, such as Gag (structural proteins), Pol, (enzymes for reverse transcription and integration into the host genome), Env, (viral envelope glycoprotein), plus the gene of interest (GOI). These genes are separated into plasmids, including the envelope plasmid, packaging and the vector of interest. For this purpose, packaging cells are used, which act as “viral particle factories”, assembling the different components of the vector and providing the virus membrane (106).

Integration of the DNA into the genome is achieved through the action of reverse transcriptase and integrase, allowing stable transduction of the T-lymphocyte and its lineage. This results in long-term expression of the transgene in lymphocytes capable of long-term survival, making CAR-Ts “living drugs” (105).

It is important to note that integration of the transgene into the genome carries the risk of oncogenesis. Lentiviruses have a preference for transcriptionally active regions, which can disrupt gene expression or inactivate tumor suppressor genes. On the other hand, gammaviruses preferentially integrate at transcriptional start sites, which increases the risk of oncogene expression. However, T-lymphocytes have been found to have a low susceptibility to transformation, making transfection with viral vectors safe in the long term. However, viral vectors may trigger an immune response against the epitopes encoded by the vector, which may limit the efficacy and persistence of the transduced cells (105), so further optimization of the system is needed.

###### 2.3.2.2.2 Non-viral

As an alternative to viral transduction, electrotransfection emerged, a technique that involves the creation of pores in the cytoplasmic membrane of the target cell by means of electrical pulses, through which the genetic material of interest is introduced. However, this technique is considered inefficient, as it requires additional systems to achieve stable transduction (105).

In this regard, transposon/transposase-based systems such as “Sleeping beauty” (SB) and “piggyBac” (PB) have emerged that have demonstrated increased efficiency in non-viral transduction. In the case of SB, the vector consists of two functional components: transposon DNA, which carries the gene of interest flanked by inverted repeat sequences (ITRs), and the SB transposase, which recognizes the ITR motifs and mobilizes the transgene to an acceptor site within the cell genome. The transposase gene is then degraded, preventing genotoxicity. On the other hand, the PB vector consists of the PB transposase (PBase), in the form of DNA or mRNA, and a separate transfer plasmid containing the genetic material of interest (105).

The great advantage of these strategies compared to viral systems is their higher loading capacity, although there is an inverse correlation between insert size and transposition efficiency. It is important to keep in mind that these strategies require the electroporation method, as nucleic acids cannot spontaneously penetrate the target cells, which decreases cell viability (105).

### 2.4 Clinical applications

CAR-T immunotherapy is an effective option for use in advanced tumors. With FDA approval, this method has revolutionized current therapy. As a highly specialized treatment, it is effective with different haematological and solid tumors, yet the associated side effects are very strong.

#### 2.4.1 Approved compounds or therapies

In 1989, a major scientific milestone was achieved by Gross et al. who succeeded in developing the first synthetic receptor expressed on lymphocytes (107). Much later, in 2017, the FDA approved the first two CAR-T-based therapies for the treatment of cancer. Specifically, approval was granted to Kymriah (tisagen lecleucel) and Yescarta (axicabtagene ciloleucel), which are used to treat patients up to 25 years of age with refractory or relapsed B-cell acute lymphoblastic leukaemia (B-ALL), and adult patients with relapsed or refractory diffuse large B-cell lymphoma (DLBCL) after two or more lines of systemic therapies (108).

Since then, several studies have been conducted to evaluate the efficacy and safety of these immunotherapies in a wide range of solid and haematological tumors (66). These efforts have resulted in the approval of six products based on this cell therapy, four of which target the anti-differentiation cluster (CD19) and the two most recent ones target the B-cell maturation antigen (BMCA) (66). These advances have had a significant impact on the treatment of diseases such as acute lymphoblastic leukaemia (ALL), B-cell neoplasms and multiple myeloma. **Supplementary Table S2** summarizes the trade names, approval dates and target antigens of these therapies.

#### 2.4.2 Clinical trials

**Figure 10:**
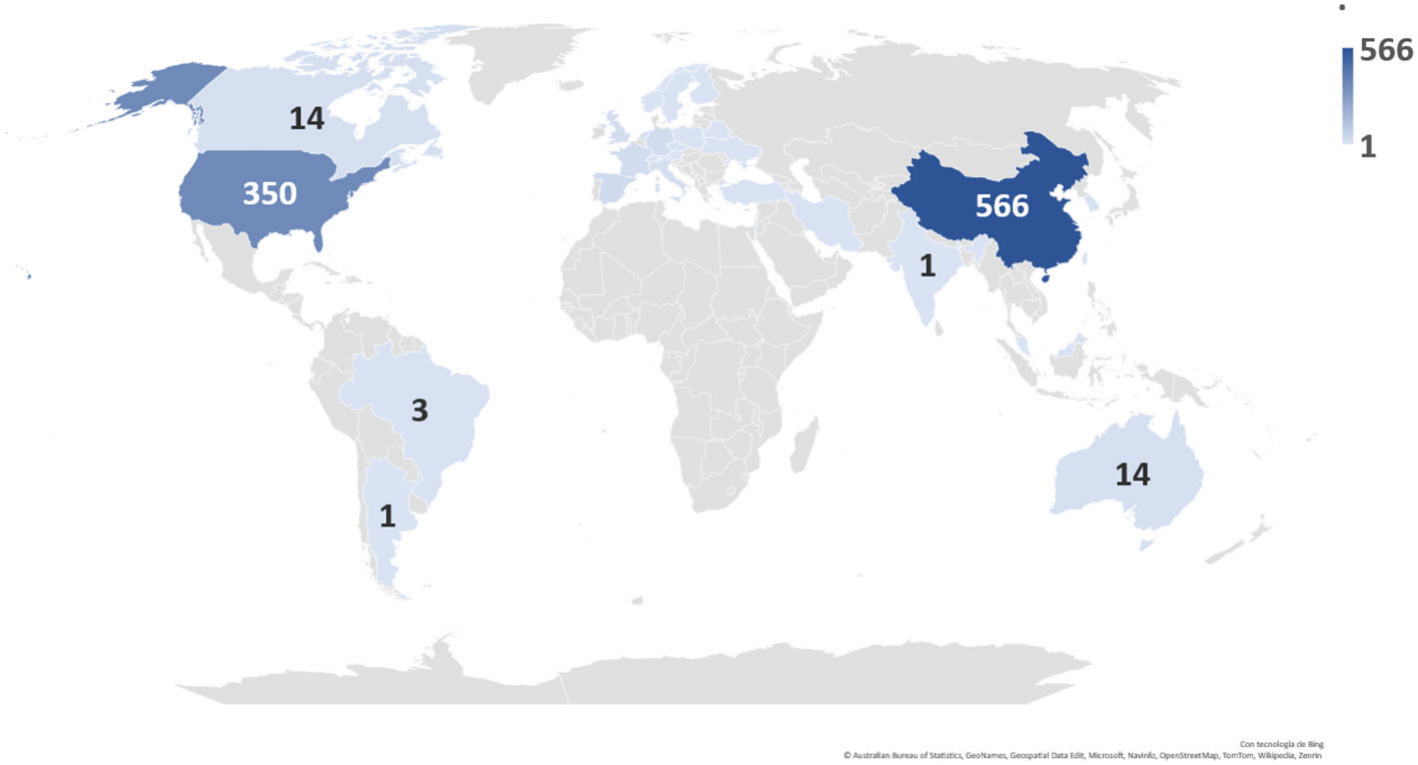
Distribution and number of registered clinical trials worldwide by country.

According to the data obtained, China was found to be the country with the highest number of clinical trials registered today, with a total of 566 ongoing clinical trials, followed by the United States (**Figure 10**).

**Figure 11:**
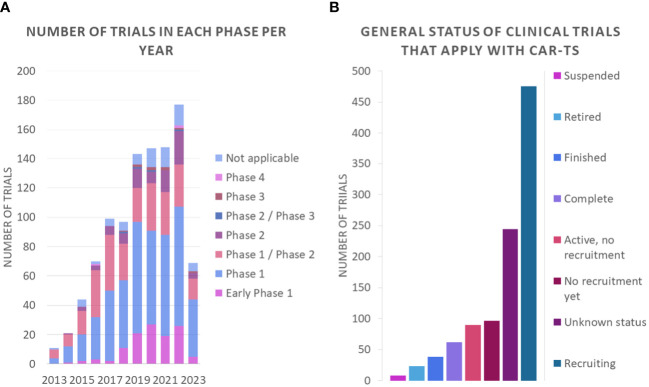
Number of clinical trials **(A)** in each phase per year and **(B)** general status of clinical trials that apply with CAR-Ts.

An analysis of the nature of these clinical trials shows that most of them are phase 1 or phase 1/phase 2 studies (**Figure 11A**). These trials focus on assessing the safety of CAR-T therapy and determining the appropriate dose of treatment. In Phase 1 studies, the safety and tolerability of treatment is investigated in a small group of participants, while Phase 1/Phase 2 studies assess both safety and preliminary efficacy in a larger group. These data suggest that promising research in the field of CAR-T therapy is ongoing, but still in early stages of development. In addition, it is important to note that most of the clinical trials are in the recruitment phase (**Figure 11B**).

**Figure 12:**
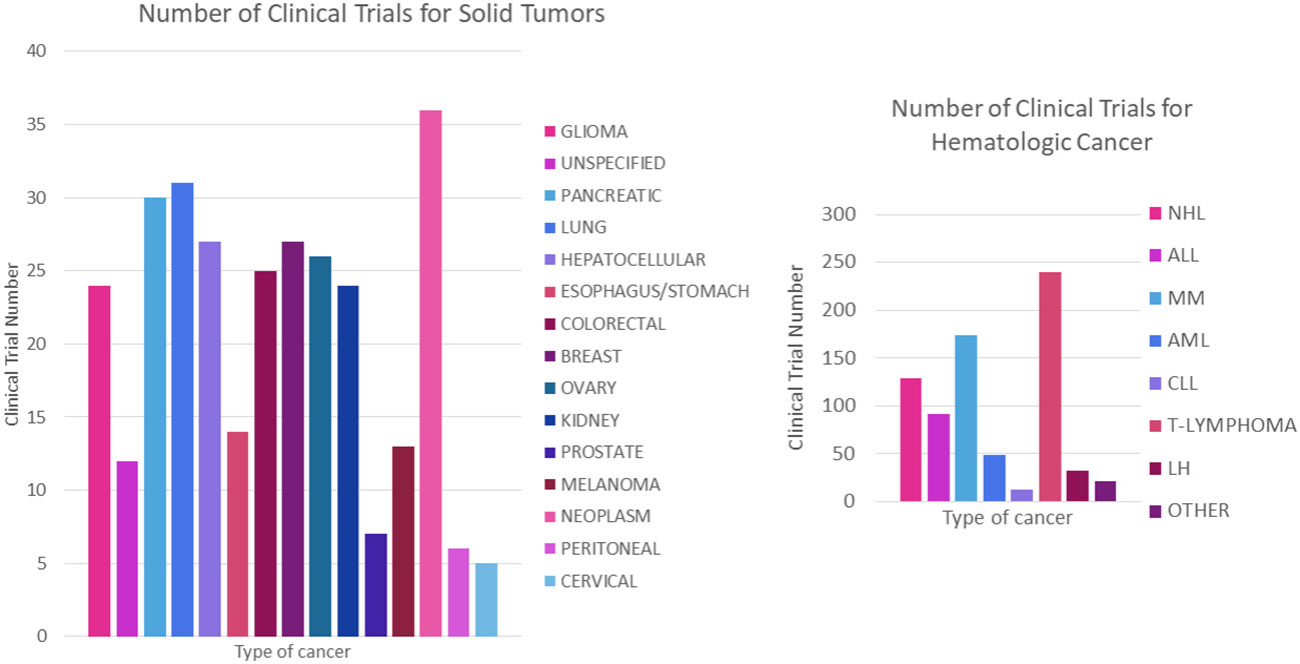
Number of registered trials for CAR-T cancer treatment. **(A)** Number of clinical trials for haematological cancer. **(B)** Number of clinical trials for solid tumors. NHL, Non- Hodgkin’s lymphoma; ALL, acute lymphoblastic leukaemia; MM, multiple myeloma; AML, acute myeloid leukaemia; CLL, chronic lymphoid leukaemia; HL, Hodgkin’s lymphoma.

It is also worth noting that clinical trials using CAR-T are more numerous for haematological cancers compared to solid tumor forming cancers [70% vs. 30% respectively (109)] which may be due to several factors, such as the ease of finding well-defined and specific cell surface markers as well as the accessibility of the tumor microenvironment compared to solid tumor forming cancers (109–112). Nevertheless, the number of trials for the latter is growing over the years, where **Figures 12A, B** show the breakdown of the number of clinical trials registered for each cancer type, giving a more detailed picture of the distribution of CAR-T therapy research.

##### 2.4.2.1 Applications in haematological malignancies

Haematological malignancies, including leukaemias and lymphomas, form a heterogeneous group of diseases arising from clonal expansion of haematopoietic cells, in bone marrow or secondary lymphoid organs. Within this group are B-cell lymphoproliferative syndromes, such as B-cell chronic lymphocytic leukaemia (B- CLL), B-cell acute lymphoblastic leukemia (B-ALL) and non-Hodgkins lymphomas (NHL). These neoplasms share widespread expression of the CD19 marker, which plays a crucial role in the survival and proliferation of malignant cells. CD19 is therefore a promising target for CAR-T immunotherapy, although elimination must be medically controlled to avoid additional risks, such as infections (113).

ALL is a rare neoplasm with 75% of cases developing from B-lymphocyte lineage precursors (B-ALL), on which we will focus. **Supplementary Table S3** summarizes the characteristics and key target antigens of CAR-T cell therapy for B-ALL.

CAR-T lymphocyte therapy targeting the CD19 antigen has been shown to be highly effective in the treatment of B-ALL, with complete remission rates between 70-90% in paediatric and adult patients (113). However, differences in outcomes have been observed depending on the type of co-stimulatory domain used in the CAR construct; for example, patients treated with 4-1BB had undetectable minimal residual disease (MRD) in contrast to patients treated with CD28 constructs (114). While CD19-targeted CAR-T therapies have produced durable remissions, potentially severe toxicities have limited their use. Moreover, since CD19-targeted CAR-Ts relapse in 10-20% of cases, due to loss of the target antigen, other CAR designs were conducted for other target antigens, such as in the case of the trial by Pan et al. (CD22) showing CR in 70-80% of patients, in addition to lower toxicities associated with CD22 CAR-Ts (114).

Chronic lymphocytic leukaemia (CLL), on the other hand, is a type of cancer characterized by the transformation and accumulation of malignant monoclonal B cells in peripheral blood and lymphoid organs. Anti-CD19 CAR-T cells have been used in patients with CLL, achieving complete remissions and the presence of CAR-T in the blood for more than 10 years. However, subsequent studies have shown limited efficacy, with a complete response rate of 30%, which is lower than that observed in ALL (115).

Currently, CAR-T therapy is also being studied for myeloproliferative disorders. For example, in patients with chronic myeloid leukemia (CML), who present survival rates of around 10% and resistance to treatment, different CAR-T strategies have been employed in pre-clinical and clinical studies (116). These studies have focused on a variety of cell-surface markers such as CD123, CVD33, Cd7, or CD70, among others. (117). Amongst these, CD123 has emerged as an especially promising target, with directed CAR-T cells capable of eliminating the cancerous cells. However, some associated toxicity has been detected, posing significant clinical concern, against which researchers have proposed a number of strategies. These include mutations in the anti-CD123 CAR-T antigen binding domain, thus reducing the affinity to healthy tissues with low CD123 expression. (118).

Cells targeting the CD33 marker have also been successful, as it is expressed in myeloid cells and in 80-90% of patients with acute myeloid leukemia (AML) (116, 119). Clinical trial NCT01864902 found a noticeable decrease in blastocysts of a patient’s bone marrow two weeks after treatment, although there was later recurrence (120). Another phase I clinical trial (NCT03018405) targeting NKG2D showed a 42% RC/ICi in patients with recurring AML (121). In addition, in order to counteract the lack of specific markers, studies have been done using dual CAR-T cells, such as those trials with CAR-T cells with simultaneous targeting of CD33 and CLL-1. This phase 1 trial was capable of reaching remission without any evidence of minimal residual disease (122).

Finally, NHL is characterized by uncontrolled proliferation of B lymphocytes in lymph nodes, bone marrow or other lymphoid tissues. The most common types of NHL include follicular lymphoma (FL) as well as DLBCL. As above, the CD19 antigen is also a target for CAR-T therapy. Patients treated with anti-CD19 CAR-T have shown durable remissions in 33-40% of treated patients (123). In addition, anti-CD20 CAR-T has been developed for refractory/relapsed patients with promising results as 2/3 of patients remained progression-free for 24 months (115).

##### 2.4.2.2 Multiple myeloma

Multiple myeloma is a disease characterized by the proliferation and accumulation of clonal plasma cells in the bone marrow with damage to other organs. Interest in developing CAR-T to treat multiple myeloma has increased dramatically in recent years, with a focus on surface antigens such as CD19 and BMCA.

Although CD19 is not directly associated with MM, it has been shown to be expressed on a minority subset of myeloma stem cells, which are associated with increased drug resistance and are responsible for the incurable nature of MM. In a clinical trial by Garfall and Maus et al. achieved a CR of 100%, despite the absence of CD19 on 99.5% of malignant cells in the only patient treated (124).

BMCA is a protein expressed on the cell surface exclusively by the B-lymphocyte lineage (80). Two CAR-T anti-BMCA therapies are currently approved: Idecabtagene Vicleucel (CR: 33% and ORR:73%) and Ciltacabtagene Autoleucel (CR:67% and ORR:100%) based on the results of the KARMMA-2 and CARTITUD-1 clinical trials, respectively. In addition, a clinical trial (EVOLVE) is ongoing for Orvacabtagene Autoleucel, with initial results putting the ORR at around 92% (125).

Despite these recent advances in treatment, disease recurrence remains a major obstacle due to the loss of molecules such as BMCA, among others, and poor CAR-T persistence caused by the tumor environment and malignant plasma cells (125).

##### 2.4.2.3 CARs in the treatment of solid tumors

**Figure 13:**
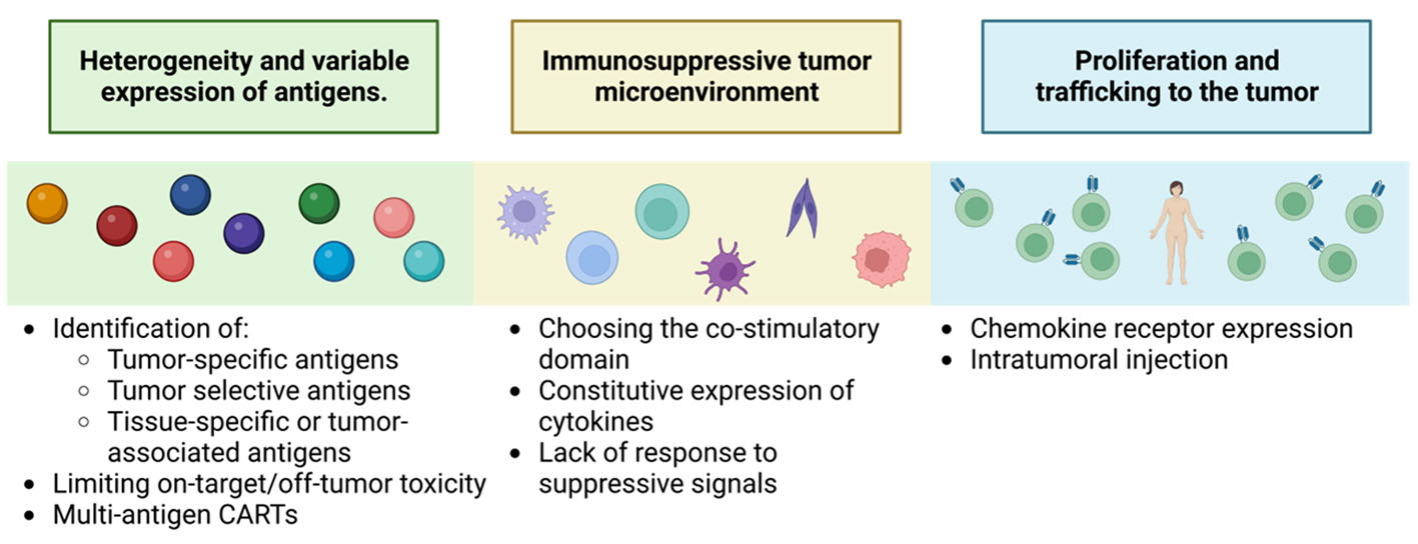
Key challenges to successful CAR-T therapy for solid tumors. Adapted from: (126).

Despite the success of CAR-T in haematological malignancies, its application in solid tumors presents additional challenges that need to be addressed (**Figure 13**).

One of the main challenges is the identification and characterization of the target antigen. Solid tumors have greater heterogeneity and variable expression of specific tumor antigens, such as proteins resulting from unique post-transcriptional modifications, like alterations in the glycosylation patterns of MUC1, MUC16, TAG72 or B7-H3 (126). In addition, TAAs are more common, which increases the risk of off-tumor toxicities in the target (127). This tumor heterogeneity also includes loss or downregulation of the expression of the antigen of interest promoting patient relapse (126).

The second limitation is CAR-T trafficking to the tumor. CAR-Ts lack the necessary corresponding chemokine receptors, which reduces their cytotoxic capacity (128). To overcome this obstacle, the development of fourth-generation CAR-Ts expressing specific chemokine receptors that match the chemokines produced by the tumor is required (128). In addition, several preclinical studies have shown greater efficacy by injecting CAR-Ts directly into the tumor (126).

Another important limitation is overcoming the immunosuppressive environment generated by tumor cells and the surrounding stroma. Studies have been performed in second- generation CAR-T that highlight the importance of the co-stimulatory domain. In addition, approaches that induce local release of stimulatory factors to promote anti-tumor immune responses are being investigated, such as TRUCKs, which represent a more promising generation for this type of treatment. In preclinical studies, work is already in progress to develop this second-generation CAR-T, specific for markers of the tumor-initiating cell population, such as CD133 (129), to demonstrate their selectivity and efficacy against refractory, highly aggressive tumors with a high rate of metastasis production. Also new strategies are using bispecific antibodies (BsAb) which, as the name suggests, are antibody constructs with the ability to bind two antigens or epitopes. Single-stranded tandem variable fragments or Bispecific T-cell Engagers (BiTE ^®^) consist of two single-stranded variable fragments connected by a flexible glycine-serine connector region. They generally target the CD3ϵ subunit of the T-cell receptor and a tumor-associated or tumor-specific antigen. Their dual specificity allows the use of BsAb in many ways, such as recruiting immune cells or blocking immune checkpoint receptors, inflammatory factors or dual signaling pathways. Most bispecific antibodies that target immune cells act by binding the CD3ϵ subunit of T cells and a tumor cell antigen to form a cytolytic synapse. This bypasses the need for MHC presentation, directly triggering activation signaling leading to target cell apoptosis, for applications also in solid tumors even in such treatment-resistant tumors as glioblastoma, (130).

Finally, cytokine signaling has been studied to enhance CAR-T cell functions and to prevent or minimize CAR-T cell-related toxicities. Another notable advance in solid tumors, such as melanoma, has been the characterization of a subset of tumor-infiltrating CD4+ T cells that express the FcγRI receptor. The receptor based FcγRI structure allows T cells to target tumor cells using intermediate antibodies. These T cells show efficient and specific cytotoxicity only when an appropriate antibody is added and their cytotoxic activity depends on the density of the target protein, thus affecting tumor cells with high antigen density, not affecting normal cells with low or no expression. This activation mechanism prevents premature depletion and, during antibody-dependent cytotoxicity, the cells secrete attenuated levels of cytokines compared to CAR-T cells, thus improving their safety profile. They have been tested in solid tumors, infiltrating the tumor microenvironment and facilitating the recruitment of host immune cells in immunocompetent mice. Unlike CAR-T therapies, which require changing the receptor in different types of cancer, FcγRI T cells serve all tumor types, changing only the injected antibody (130).

##### 2.4.2.4 CARs in the treatment of non tumoral diseases.

Anything that has the capacity to restructure the immune system in T-lymphocytes may have the possibility of application. This includes autoimmune (131), as lupus, nephrological diseases such as systemic vasculitis or neurological diseases such as multiple sclerosis. Its results have already been proven in viral and fungal infections, as well as in multiple types of diabetes, thyroid, etc.

Systemic lupus erythematosus (SLE) is a life-threatening autoimmune disease characterized by adaptive activation of the immune system, formation of double-stranded DNA autoantibodies and organ inflammation. Despite advances in the treatment of SLE, some patients fail to respond to current state-of-the-art therapies and are at high risk of organ failure and even death. The results of Schett and coworkers (132, 133) show the use of CAR-T against CD19, lupus remission of up to 17 months is posible. Adoptive immunotherapy with regulatory T cells (Tregs) is emerging as a viable drug therapy option for various inflammatory states and autoimmune and alloimmune diseases (134–136).

### 2.5 General limitations or side effects and traditional treatments

The main toxicities of CAR-transduced T cells fall into two categories: general toxicities, such as cytokine release syndrome (CRS) and immune effector cell-associated neurotoxicity (ICANS); and specific toxicities due to the interaction of CAR with antigens expressed on healthy cells, known as off-tumor on-target toxicities, mentioned above (Section 2.3.2.1).

#### 2.5.1 Cytokine release syndrome

CRS is the most common toxic side effect of CAR-T therapy. For example, in haematologic malignancies treated with CD19-targeted CAR-T with Blinatumomab (the first bispecific T-cell-engaging single-chain antibodies), close to 100% of patients develop CRS (137). It consists of a systemic inflammatory response caused by significant cytokine up-regulation accompanied by rapid *in vivo* activation of CAR-Ts after the initial infusion. Binding of the scFv domain of CAR-T to the target antigen triggers the proliferation and secretion of high amounts of cytokines such as IL-6, IFN-γ, IL-10 and TNF-α in a short period of time. In turn, several studies have shown that IFN-γ induces the release of other cytokines such as TNF-α, IL- 6, IL-15, IL-1β and IL-12, maintaining the above responses through positive feedback (138). CRS can present a wide range of symptoms, from mild (flu-like) to severe and life threatening. Mild symptoms include fever, fatigue, rash, headache, arthralgia and myalgia, while severe cases are characterized by hypotension and high fever and may progress to uncontrolled systemic inflammatory responses such as circulatory shock, coagulopathies and multiorgan failure, which can result in death. Common laboratory abnormalities in these patients include cytopenias, elevated creatinine and liver enzymes, and altered coagulation parameters, among others (137). All this symptomatology causes patients to develop an initial low-grade CRS that, unfortunately, may progress to a high-grade CRS with severe outcome. Currently, treatment of CRS caused by CAR T-cell therapy is limited to tocilizumab (TCZ) and corticosteroids in clinical guidelines, and also glycyrrhizin has therapeutic potential for treating CRS caused by CAR T-cell therapy (139).

Other treatments being used to alleviate these effects produced by the cytokine storm include the use of different drugs such as Glycyrrhizin (139), a pleiotropic molecule that is well tolerated and affordably priced. The interleukin-6 receptor antibody tocilizumab has been used for advanced CRS prior to the use of corticosteroids (140). Prophylactic tocilizumab (1 hour prior to infusion of anti-CD19 CAR-T cells in patients with non-Hodgkin’s lymphoma) has been also associated with a low incidence and severity of CRS, with no detected adverse effects and no increase in the frequency or severity of neural pathology. These contributions proclaim excellent disease control and overall survival as applied to non-Hodgkin’s lymphoma patients (141). And although corticosteroids were initially applied at advanced stages of CRS complication, recently there have been contributions pointing to the benefits of early use of corticosteroids after CAR T-cell therapy to reduce the risk of high-grade CRS in patients with initial CRS. This treatment does not seem to have a negative impact on other parallel side effects such as neurotoxicity or treatment outcome (142).

#### 2.5.2 Immune-associated effector cell-associated neurotoxicity

CAR-T cell-related encephalopathy syndrome (CRES) is alternatively known as immune effector cell-associated neurotoxicity syndrome (ICANS). To avoid the systemic toxicities mentioned above and achieve optimal clinical efficacy, it is necessary to reach a threshold level of cytokine activation and secretion tolerable to the body in CAR-Ts. This is achieved by correct dose escalation in phase I trials for each type of CAR, taking into account overall tumor burden, target antigen expression levels and ectodomain affinity, as well as co-stimulatory domains (83).

Other treatments, such as Anakinra, are feasible and safe for grade ≥2 ICANS improvement of symptoms despite treatment with high-dose corticosteroids (143), although a limited impact on CAR-T efficacy is observed.

### 2.6 Novel safety strategies to eliminate CAR-T-generated toxicities

#### 2.6.1 Suicide gene control

**Figure 14:**
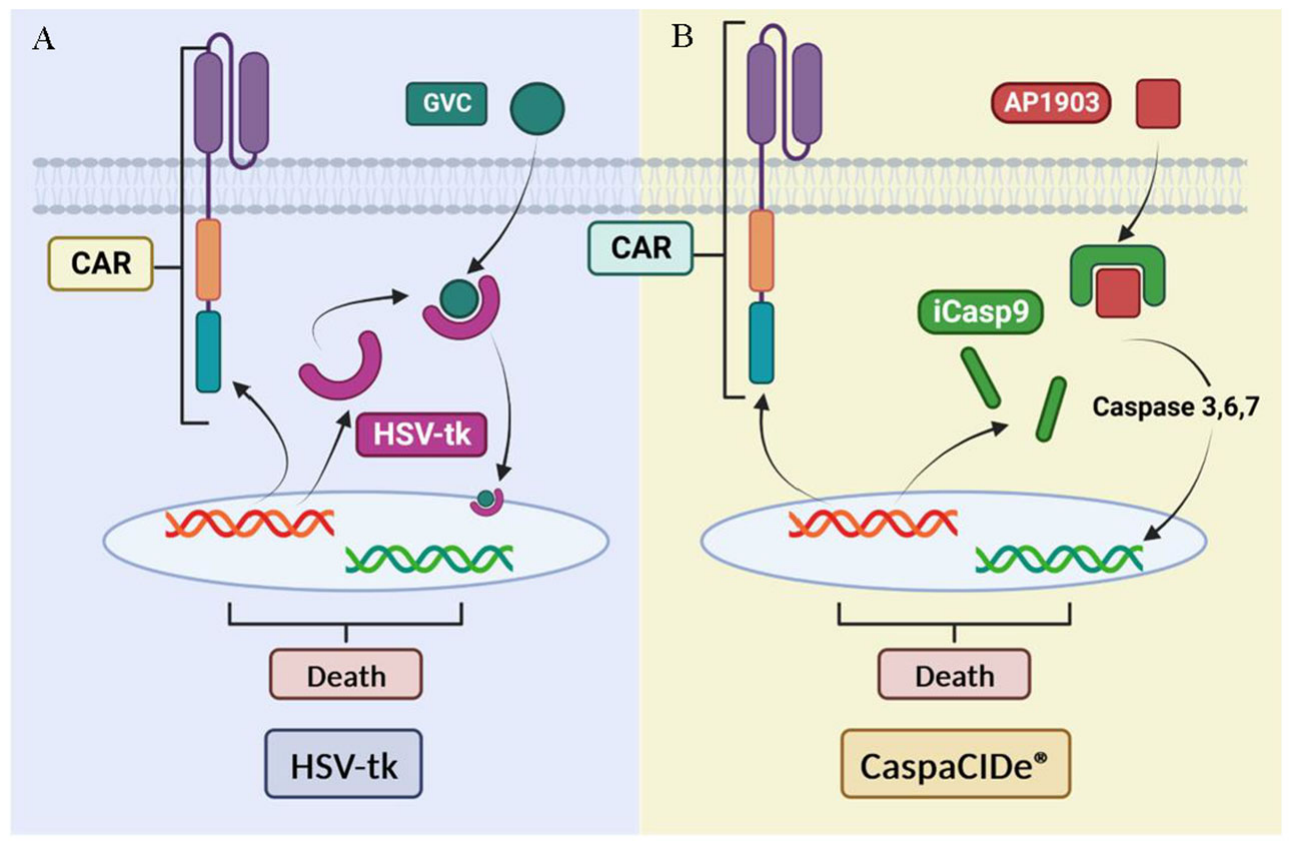
Suicide gene-based security systems: **(A)** HSV- tk-based system. **(B)** iCasp9-based system. Adapted from: (144).

Safety strategies have been developed to address CAR-T toxicities, such as the use of suicide genes (**Figure 14**). Suicide genes are designed to program cell death to selectively eliminate a certain population of cells in an organism using specific signals, designated the safe-cell system (145) Their use allows the elimination of therapeutic cells in the face of the unpredictable expansion of these cells and avoids toxicity in patients (146) with the main objectives of the control of proliferation and improvement of biosafety. This also prevents the uncontrolled release of pro-inflammatory cytokines into the bloodstream and its inflammatory response, using these genes as switches (147). However, we also highlight the adverse effects of suicide genes in CAR-T therapy, including treatment interruption and cytokine release syndrome by this abrupt interruption.

One example of these safety strategies is herpes simplex virus thymidine kinase (HSV-tk), which phosphorylates specific nucleoside analogues to form a toxic compound (GCV triphosphate) that inhibits DNA synthesis and causes cell death described for 20 years (148). The system has been previously validated in the control of induced stem cell therapies to improve their biosafety (149). Although HSV-tk expressing cells have demonstrated high anti-tumor efficacy and can be effectively killed, this approach has limitations such as the need for activation by a prodrug (ganciclovir) and a time of 3 days to achieve full effect *in vitro* (144).

Another alternative suicide gene system is CaspaCIDe^®^, which uses the inducible safety switch gene caspase 9 (iCasp9). This system, previously used for MSC anti-cancer treatments elimination (150), enables the elimination of inappropriately activated CAR-T by using a small-molecule dimerization-inducing drug called AP1903, which promotes apoptosis (151) in transduced cells. This gene is composed of the intracellular portion of the human caspase 9 protein fused to a drug-binding domain derived from the human FK506 protein. The CaspaCIDe^®^ system is able to selectively kill cells expressing high levels of the suicide gene (152). This mechanism is implemented to effectively control adverse events in patients who have received a hematopoietic stem cell transplant, a common procedure in the treatment of haematological diseases and certain types of cancer. The main function of the iC9 is to manage possible unwanted side effects or adverse reactions that could arise after transplantation, thus providing an additional layer of safety in the treatment process (153).

A problem that has also been highlighted and is being attempted to be addressed is related to the underlying mechanisms of T cell exhaustion. The American group of Diana Gumber and Leo D. Wang present a summary of emerging strategies to prevent or reverse depletion through modifications of the CAR receptor or CAR-independent pathways. These strategies have broad potential to improve the clinical outcomes of CAR T cell therapy (154).

Preclinical research shows the possibility of integrating these genes to eliminate cells in case of severe toxicity and, in this way, modulate the activity of CAR-T cells. However, one of the limitations of the use of suicide genes is that it affects the therapy of rapidly progressing diseases in an abrupt manner. The group of Mestermann et al. have identified the drug dasatinib as a temporary inactivator of CAR-T cells. This contributes to reducing acute toxicity, allowing T cells to recover their antitumor effects after the drug is withdrawn, that is, reversible inhibition by putting CAR-T cells into inactivity (155). Since continuity of CAR T therapy is crucial to maintain treatment efficacy and control rapidly growing diseases, definitive discontinuation of CAR T therapy is avoided as it would mean loss of treatment control and efficacy. One of the most important challenges in around 50% of cases is recurrent diseases with changes in the expression of target antigens. This is due to genetic alterations, immune edits, clonal selection, and antigen elimination. Gupta et al. propose a drop of up to 57% in antigen expression in patients with CAR-T therapy and an uncontrolled release of cytokines (156). The release of antigens by CAR-T cells can trigger unwanted responses and produce systemic inflammation. The activation of suicidal genes can also produce this process, which triggers cytokine release syndrome, producing toxicity, haematological alterations and damage to multiple organs. Zhao et al, explain the adverse events associated with therapy using ocular models and a high expression of cytokine release syndrome (157). Rejeski et al, similarly, explain the inflammatory changes capable of inducing CART expansion dynamics, which produces haematotoxicity (158). The release of cytokines can be classified according to the reaction it is capable of triggering, where different pathophysiologies are shown, including the most serious, death (159).

#### 2.6.2 Alternative method to gene manipulation with “suicide” genes, for CAR-T regulations

Other known methods are the cell elimination system by recognition of CD20 or EGFRt membrane proteins mediated by NK cells (160), representing an alternative method to gene manipulation with “suicide” genes (161). Ruxolitinib reduces severe CRS response by suspending CAR-T cell function instead of damaging CAR-T cells. Ruxolitinib significantly reduced the proliferation rate of CAR-T cells *in vivo* without affecting the therapeutic efficacy after withdrawal at the appropriate dose (162).

The tetracycline-inducible system corresponds to one of the most widely used methods to efficiently control cell proliferation through the use of gene expression with the antibiotic doxycycline (163). Primary T cells obtained by this treatment show no apparent functional changes (164). These advances show tremendous clinical results with high therapeutic efficacy already proven in *in vitro* and *in vivo* models (R.-Y. 165). Other strategies, such as control by small molecules, allow simple and rapid control with high kinetics and cellular diffusion. Examples are natural resveratrol compounds, drugs such as endoxifen or tamoxifen, which aim to precisely control the expression of CAR-T markers (166). These systems allow the conditional activation of CAR-T to the presence of molecules such as tamoxifen, so that in the absence of tamoxifen, side effects cease (167).

And even the activity of intratumoral CAR-T cells can be controlled photothermally via synthetic gene switches that trigger the expression of transgenes in response to mild temperature elevations (to 40–42°C) (168).

Despite the effectiveness of eliminating CAR-T cells, there are alternatives capable of regulating their activity and expression level in order not to permanently lose these cells. For this purpose, antigen expression monitoring technologies have been developed using NGS [(169), multimodal diagnostic tools, such as liquid biopsy, are being (170) and multiplexed assays (171–173)]. Finally, and just as importantly, biosafety management is still under study to control therapeutic cells, CAR gene expression and control their localization, prevent immune cell exhaustion, limit activities in unwanted areas, and improve the modular precision of antigens, which remain significant challenges (174). It is still necessary to optimize strategies and find a balance between patient monitoring and continued treatment effectiveness.

A new approach from “off the shelf” allogeneic CAR-T cells emerges to potentially address problems of autologous CAR-T with a customised manufacturing process for each patient (high cost, minimum production time of 3 weeks, and deficient number of functional T lymphocytes in peripheral blood possibly due to side effects of the patient’s previous treatments) (175), as they have several potential advantages, such as reduced cost due to the implementation of industrialised processes, where CAR-T can be performed for several patients from a single donor, as well as generating a cryopreserved product (176), making the treatment immediately available to patients who require it. This can be a significant challenge in patients with highly proliferative disease and there are already numerous clinical trials validating its efficacy (177).

However, they also have a number of immunological drawbacks, as they can cause life-threatening graft-versus-host disease (GVHD). In addition, these allogeneic T cells can be rapidly eliminated by the patient’s own immune system, limiting their persistence and anti-tumour efficacy. The T cells used in CAR-T therapy can come from a variety of sources, such as peripheral blood (PBMC) or umbilical cord blood (UCB), and even embryonic or induced pluripotent stem cells (iPSCs). The latter offer the advantage of being clonally homogenous and can be genetically edited to minimise GVHD (177).

A key strategy to mitigate GVHD involves genetic modification of T cells to block αβ-TCR expression or signalling. Disruption of the TRAC gene, which encodes the alpha chain of the TCR, has been identified as an effective method to prevent GVHD without compromising the anti-tumour efficacy of CAR-T cells (178, 179)This technique has been applied in clinical trials, as in the case of the UCART19 product, a product that involves the use of transcription activator-like nucleases (TALEN) to simultaneously knock out TRAC and CD52 genes, observing 64% knockout in CAR-T cells, without causing severe GVHD in treated patients (180.

There are also new technological advances in order to reduce costs and preparation time. New CARs are being designed with a modular or splitting domain approach, so that the antigen recognition domain is separated from the signaling domain of a conventional CAR, so that the target antigen can be more easily changed or redirected without the need to redesign the CAR T cells. Therefore, this CAR system can serve as a universal CAR (UniCAR) (181).

Finally note that to overcome the obstacles of CAR-T therapy in clinical treatment, emerging cell-free therapies based on CAR-T cell-derived exosomes have been developed as an effective and promising alternative approach (182).

## 3 Conclusions

- CAR-T cells have emerged as an innovative and promising strategy in the field of cancer immunotherapy, and throughout this literature review the therapeutic potential of this therapy has been thoroughly explored, investigating its structure, anti-tumor efficacy, side effects and production, as well as its remaining limitations and challenges in clinical application. This final section lists the conclusions drawn from the review and provides future perspectives and recommendations for improving and enhancing CAR-T lymphocyte therapy.
- CAR-Ts have high applicability as they could be used in a wide range of cancer types as long as tumor-associated or tumor-specific antigens are found to be expressed on the cell surface and their targeting is not toxic to the patient.
- CAR-T-based therapy can generate durable responses in cancer patients, as optimization of the CAR structure across generations has improved the persistence of these cells in the body and their ability to eliminate residual tumor cells, thus contributing to prolonged remission and long-term survival. However, potential relapses associated with loss of tumor antigen or tumor escape mechanisms must be considered.
- Targeting single antigens can lead to the risk of immune escape by the tumor, which can be reduced by targeting multiple antigens (87), for example, the bi-specific CARs HER2/IL13Ra2 (glioblastoma) and HER2/MUC1 (breast cancer) have been shown to produce superior anti-tumor responses compared to single-target therapy (87, 183).
- CAR-T lymphocytes have proven to be a promising strategy in the treatment of certain types of cancer, especially in haematological malignancies, achieving complete remissions of 70% ALL. However, in solid tumors the success rate is lower, as the various limitations of this therapy, such as the tumor microenvironment or the antigenic heterogeneity mentioned above, still need to be addressed.
- Despite the remarkable benefits of this therapy, associated toxicities such as systemic inflammatory responses, known as cytokine release syndrome, as well as neurotoxicity must be taken into account. These side effects must be properly monitored so as not to put the patient’s life at risk and to ensure the patient’s safety.

## 4 Future directions

Decades of work in the fight against cancer have led to significant advances in curing tumors, but success is closely linked to early diagnosis. In patients with advanced cancer, complete remission rates have hardly improved. Further research is important and the following future perspectives to improve therapy and achieve the desired success are proposed.

- The broadening of the spectrum of antigens available to treat various types of cancer requires the successful identification and characterization of the antigen to be targeted by CAR-T lymphocytes, e.g. by computer modelling (*in silico* assays).
- Improvements in persistence and durability: To this end, several trials are underway that combine various types of treatments alongside CAR-T therapy such as chemotherapy, or other immunotherapies such as checkpoint inhibitors. For example, co-administration of chemotherapy with CAR-T lymphocytes inhibits autoimmunity and immunosuppressive cells to improve CAR-T persistence *in vivo* (184).
- Improvements in security: optimizing the structure of the chimeric antigen receptor as well as introducing elements that help control security such as inducible suicide genes.
- An important area for future research is the study of the structure of the chimeric antigen receptor with the aim of creating the perfect construct adapted to each type of cancer. As we have seen, the appropriate selection of co-stimulatory domains, optimization of affinity and stability, and consideration of tumor antigen specificity are key aspects. In addition, the inclusion of safety features and combination with other therapies could further enhance their effectiveness. As genetic engineering and precision medicine advance, more sophisticated and targeted CARs are expected in the future, opening up new possibilities in personalized cancer treatment.
- Development of a greater number of allogeneic therapies (universal CAR-T), this could help us to shorten the waiting times suffered by the patient before receiving the treatment or allow entry to those cases in which they do not have enough lymphocytes required to carry out the treatment but taking into account the risks of rejection as well as reducing the cost of manufacturing.

Ultimately, it is hoped that these findings will be a starting point for future research and development in the field of cancer immunotherapy, with the aim of maximizing efficacy, minimizing toxicities, and broadening the scope of CAR-T lymphocytes as an innovative and promising therapeutic strategy in the fight against cancer.

## Author contributions

PE: Conceptualization, Data curation, Formal analysis, Investigation, Methodology, Resources, Validation, Writing – original draft, Writing – review & editing. MS: Data curation, Project administration, Writing – review & editing. NA: Data curation, Writing – review & editing. JQ: Data curation, Project administration, Writing – review & editing. CG: Data curation, Writing – review & editing. RG-M: Data curation, Funding acquisition, Supervision, Validation, Writing – review & editing. CR: Conceptualization, Funding acquisition, Project administration, Resources, Supervision, Validation, Visualization, Writing – review & editing.

## References

[1] Molnar C Gair J. 6.3 Cancer and the Cell Cycle. Canada: BCcampus (2015).

[2] Gil-Gas C Sánchez-Díez M Honrubia-Gómez P Sánchez-Sánchez JL Alvarez-Simón CB Sabater S. Self-renewal inhibition in breast cancer stem cells: moonlight role of PEDF in breast cancer. Cancers. (2023) 15:5422. doi: 10.3390/cancers15225422 38001682 PMC10670784

[3] Kuburich NA den Hollander P Castaneda M Pietilä M Tang X Batra H. Stabilizing vimentin phosphorylation inhibits stem-like cell properties and metastasis of hybrid epithelial/mesenchymal carcinomas. Cell Rep. (2023) 42:113470. doi: 10.1016/j.celrep.2023.113470 37979166 PMC11062250

[4] Yadav RP Baranwal S. Kindlin-2 regulates colonic cancer stem-like cells survival and self-renewal *via* Wnt/β-catenin mediated pathway. Cell Signalling. (2024) 113:110953. doi: 10.1016/j.cellsig.2023.110953 38084837

[5] Sung H Ferlay J Siegel RL Laversanne M Soerjomataram I Jemal A. Global cancer statistics 2020: GLOBOCAN estimates of incidence and mortality worldwide for 36 cancers in 185 countries. CA: A Cancer J Clin. (2021) 71:209–49. doi: 10.3322/caac.21660 33538338

[6] Cancer today. Available online at: https://gco.iarc.fr/today/home.

[7] Fedewa SA Sauer AG Siegel RL Jemal A. Prevalence of major risk factors and use of screening tests for cancer in the United States. Cancer Epidemiology Biomarkers Prevention : A Publ Am Assoc Cancer Research Cosponsored by Am Soc Prev Oncol. (2015) 24:637–52. doi: 10.1158/1055-9965.EPI-15-0134 25834147

[8] Podolskiy DI Gladyshev VN. Intrinsic versus extrinsic cancer risk factors and aging. Trends Mol Med. (2016) 22:833–4. doi: 10.1016/j.molmed.2016.08.001 PMC5068914 27544777

[9] Yi P Yu W Xiong Y Dong Y Huang Q Lin Y. Interleukin 35: new target for immunotherapy targeting the tumor microenvironment. Mol Cancer Ther. (2023) 166:115336–115336. doi: 10.1158/1535-7163.MCT-23-0242 37988561

[10] Zhang J Liu S Chen X Xu X Xu F. Non-immune cell components in tumor microenvironment influencing lung cancer Immunotherapy. Biomedicine Pharmacotherapy. (2023) 166:115336. doi: 10.1016/j.biopha.2023.115336 37591126

[11] Zhao Y Du J Shen X. Targeting myeloid-derived suppressor cells in tumor immunotherapy: Current, future and beyond. Front Immunol. (2023) 14:1157537. doi: 10.3389/fimmu.2023.1157537 37006306 PMC10063857

[12] Fu T Dai L-J Wu S-Y Xiao Y Ma D Jiang Y-Z. Spatial architecture of the immune microenvironment orchestrates tumor immunity and therapeutic response. J Hematol Oncol. (2021) 14:98. doi: 10.1186/s13045-021-01103-4 34172088 PMC8234625

[13] Burnet FM. Immunological surveillance in neoplasia. Transplant Rev. (1971) 7:3–25. doi: 10.1111/j.1600-065X.1971.tb00461.x 5146537

[14] Yuan S Zhu T Wang J Jiang R Shu A Zhang Z. miR-22 promotes immunosuppression *via* activating JAK/STAT3 signaling in cutaneous squamous cell carcinoma. Carcinogenesis. (2023) 44:549–61. doi: 10.1093/carcin/bgad055 37466677

[15] Gubin MM Vesely MD. Cancer immunoediting in the era of immuno-oncology. Clin Cancer Research : Off J Am Assoc Cancer Res. (2022) 28:3917–28. doi: 10.1158/1078-0432.CCR-21-1804 PMC9481657 35594163

[16] Bartneck J Hartmann A-K Stein L Arnold-Schild D Klein M Stassen M. Tumor-infiltrating CCR2+ inflammatory monocytes counteract specific immunotherapy. Front Immunol. (2023) 14:1267866. doi: 10.3389/fimmu.2023.1267866 37849753 PMC10577317

[17] Yu H Shi T Yao L Xu D Ding Y Xia Q. Elevated nuclear PIGL disrupts the cMyc/BRD4 axis and improves PD-1 blockade therapy by dampening tumor immune evasion. Cell Mol Immunol. (2023) 20:867–80. doi: 10.1038/s41423-023-01048-3 PMC10387471 37280393

[18] Liu B Cao Y Li Y Ma H Yang M Zhang Q. Glioma stem cells upregulate CD39 expression to escape immune response through SOX2 modulation. Cancers. (2022) 14:783. doi: 10.3390/cancers14030783 35159053 PMC8834269

[19] Zhang L Kuca K You L Zhao Y Musilek K Nepovimova E. Signal transducer and activator of transcription 3 signaling in tumor immune evasion. Pharmacol Ther. (2022) 230:107969. doi: 10.1016/j.pharmthera.2021.107969 34450232

[20] Niu N Shen X Zhang L Chen Y Lu P Yang W. Tumor cell-intrinsic SETD2 deficiency reprograms neutrophils to foster immune escape in pancreatic tumorigenesis. Advanced Sci. (2023) 10. doi: 10.1002/advs.202202937 PMC9839845 36453584

[21] Eskandari-Malayeri F Rezaei M. Immune checkpoint inhibitors as mediators for immunosuppression by cancer-associated fibroblasts: A comprehensive review. Front Immunol. (2022) 13:996145. doi: 10.3389/fimmu.2022.996145 36275750 PMC9581325

[22] Raza S Rajak S Tewari A Gupta P Chattopadhyay N Sinha RA. Multifaceted role of chemokines in solid tumors: From biology to therapy. Semin Cancer Biol. (2022) 86:1105–21. doi: 10.1016/j.semcancer.2021.12.011 PMC7613720 34979274

[23] Chen DS Mellman I. Oncology meets immunology: the cancer-immunity cycle. Immunity. (2013) 39:1–10. doi: 10.1016/j.immuni.2013.07.012 23890059

[24] Alanko J Uçar MC Canigova N Stopp J Schwarz J Merrin J. CCR7 acts as both a sensor and a sink for CCL19 to coordinate collective leukocyte migration. Sci Immunol. (2023) 8. doi: 10.1126/sciimmunol.adc9584 37656776

[25] Cuenca-Escalona J Flórez-Grau G van den Dries K Cambi A de Vries IJM. PGE2-EP4 signaling steers cDC2 maturation towards the induction of suppressive T cell responses. Eur J Immunol. (2023) 541:321–30. doi: 10.1002/eji.202350770 38088451

[26] Jiang Y Hu Y Yang Y Yan R Zheng L Fu X. Tong-Xie-Yao-Fang promotes dendritic cells maturation and retards tumor growth in colorectal cancer mice with chronic restraint stress. J Ethnopharmacology. (2024) 319:117069. doi: 10.1016/j.jep.2023.117069 37619860

[27] Chen DS Mellman I. Elements of cancer immunity and the cancer-immune set point. Nature. (2017) 541:321–30. doi: 10.1038/nature21349 28102259

[28] Mittrücker H-W Visekruna A Huber M. Heterogeneity in the differentiation and function of CD8^+^ T cells. Archivum Immunologiae Therapiae Experimentalis. (2014) 62:449–58. doi: 10.1007/s00005-014-0293-y 24879097

[29] Durgeau A Virk Y Corgnac S Mami-Chouaib F. Recent advances in targeting CD8 T-cell immunity for more effective cancer immunotherapy. Front Immunol. (2018) 9:14. doi: 10.3389/fimmu.2018.00014 29403496 PMC5786548

[30] Chen Y Sun J Liu J Wei Y Wang X Fang H. Aldehyde dehydrogenase 2-mediated aldehyde metabolism promotes tumor immune evasion by regulating the NOD/VISTA axis. J ImmunoTherapy Cancer. (2023) 11:e007487. doi: 10.1136/jitc-2023-007487 PMC10711917 38088186

[31] Li B Wang B. USP7 enables immune escape of glioma cells by regulating PD-L1 expression. Immunol Investigations. (2022) 51:1921–37. doi: 10.1080/08820139.2022.2083972 35852892

[32] Bredel D Tihic E Mouraud S Danlos F-X Susini S Aglave M. Immune checkpoints are predominantly co-expressed by clonally expanded CD4+FoxP3+ intratumoral T-cells in primary human cancers. J Exp Clin Cancer Res. (2023) 42:333. doi: 10.1186/s13046-023-02897-6 38057799 PMC10699039

[33] Jiménez-Morales S Aranda-Uribe IS Pérez-Amado CJ Ramírez-Bello J Hidalgo-Miranda A. Mechanisms of immunosuppressive tumor evasion: focus on acute lymphoblastic leukemia. Front Immunol. (2021) 12:737340. doi: 10.3389/fimmu.2021.737340 34867958 PMC8636671

[34] Zhu X Liang R Lan T Ding D Huang S Shao J. Tumor-associated macrophage-specific CD155 contributes to M2-phenotype transition, immunosuppression, and tumor progression in colorectal cancer. J ImmunoTherapy Cancer. (2022) 10:e004219. doi: 10.1136/jitc-2021-004219 PMC9476138 36104099

[35] Gee AP Bruce KM van Hilten J Siden EJ Braylan RC Bauer PC. Selective loss of expression of a tumor-associated antigen on a human leukemia cell line induced by treatment with monoclonal antibody and complement2. JNCI: J Natl Cancer Institute. (1987) 78:29–35. doi: 10.1093/jnci/78.1.29 3467127

[36] Browning MJ Krausa P Rowan A Hill AB Bicknell DC Bodmer JG. Loss of human leukocyte antigen expression on colorectal tumor cell lines. J Immunotherapy. (1993) 14:163–8. doi: 10.1097/00002371-199310000-00001 8297898

[37] Hiatt K Ingram DA Huddleston H Spandau DF Kapur R Clapp DW. Loss of the nf1 tumor suppressor gene decreases fas antigen expression in myeloid cells. Am J Pathol. (2004) 164:1471–9. doi: 10.1016/S0002-9440(10)63233-6 PMC1615352 15039234

[38] Khong HT Wang QJ Rosenberg SA. Identification of multiple antigens recognized by tumor-infiltrating lymphocytes from a single patient: tumor escape by antigen loss and loss of MHC expression. J Immunotherapy. (2004) 27:184–90. doi: 10.1097/00002371-200405000-00002 PMC2275330 15076135

[39] Schuster SJ Huw L-Y Bolen CR Maximov V Polson AG Hatzi K. Loss of CD20 expression as a mechanism of resistance to mosunetuzumab in relapsed/refractory B-cell lymphomas. Blood J. (2023) 90:2390–7. doi: 10.1182/blood.2023022348 PMC10934296 38048694

[40] Zeidler R Eissner G Meissner P Uebel S Tampé R Lazis S. Downregulation of TAP1 in B lymphocytes by cellular and Epstein-Barr virus-encoded interleukin-10. Blood. (1997) 90:2390–7. doi: 10.1182/blood.V90.6.2390 9310490

[41] Kroemer G Galassi C Zitvogel L Galluzzi L. Immunogenic cell stress and death. Nat Immunol. (2022) 23:487–500. doi: 10.1038/s41590-022-01132-2 35145297

[42] Lin RA Lin JK Lin SY. Mechanisms of immunogenic cell death and immune checkpoint blockade therapy. Kaohsiung J Med Sci. (2021) 37:448–58. doi: 10.1002/kjm2.12375 PMC11896493 33636043

[43] Calvillo-Rodríguez KM Lorenzo-Anota HY Rodríguez-Padilla C Martínez-Torres AC Scott-Algara D. Immunotherapies inducing immunogenic cell death in cancer: insight of the innate immune system. Front Immunol. (2023) 14:1294434. doi: 10.3389/fimmu.2023.1294434 38077402 PMC10701401

[44] Siegel RL Miller KD Wagle NS Jemal A. Cancer statistics 2023. CA: A Cancer J Clin. (2023) 73:17–48. doi: 10.3322/caac.21763 36633525

[45] Ghiringhelli F Apetoh L Tesniere A Aymeric L Ma Y Ortiz C. Activation of the NLRP3 inflammasome in dendritic cells induces IL-1β–dependent adaptive immunity against tumors. Nat Med. (2009) 15:1170–8. doi: 10.1038/nm.2028 19767732

[46] Tang D Kang R Berghe T Vandenabeele P Kroemer G. The molecular machinery of regulated cell death. Cell Res. (2019) 29:347–64. doi: 10.1038/s41422-019-0164-5 PMC6796845 30948788

[47] Met Ö. Jensen KM Chamberlain CA Donia M Svane IM. Principles of adoptive T cell therapy in cancer. Semin Immunopathology. (2019) 41:49–58. doi: 10.1007/s00281-018-0703-z 30187086

[48] Fehleisen. Ueber die Züchtung der Erysipelkokken auf künstlichem Nährboden und ihre Uebertragbarkeit auf den Menschen. Deutsche Medizinische Wochenschrift. (1882) 8:553–4. doi: 10.1055/S-0029-1196806

[49] Busch W. Aus der Sitzung der medicinischen Section vom 13 November 1867. Berl Klin Wochenschr. (1868) 5:137. doi: 10.1177/1534735416649916

[50] Orange M Reuter U Hobohm U. Coley’s lessons remembered: augmenting mistletoe therapy. Integr Cancer Therapies. (2016) 15:502–11. doi: 10.1177/1534735416649916 PMC5739169 27207233

[51] NAUTS HC FOWLER GA BOGATKO FH. A review of the influence of bacterial infection and of bacterial products (Coley’s toxins) on Malignant tumors in man; a critical analysis of 30 inoperable cases treated by Coley’s mixed toxins, in which diagnosis was confirmed by microscopic examination selected for special study. Acta Med Scandinavica Supplementum. (1953) 276:1–103. doi: 10.3389/fimmu.2019.02965 13039964

[52] Dobosz P Dzieciątkowski T. The intriguing history of cancer immunotherapy. Front Immunol. (2019) 10:2965. doi: 10.3389/fimmu.2019.02965 31921205 PMC6928196

[53] Burdick CG. WILLIAM BRADLEY COLEY 1862-1936. Ann Surg. (1937) 105:152–5. doi: 10.1097/00000658-193701000-00015 PMC1390301 17856903

[54] Zhang Y Zhang Z. The history and advances in cancer immunotherapy: understanding the characteristics of tumor-infiltrating immune cells and their therapeutic implications. Cell Mol Immunol. (2020) 17:807–21. doi: 10.1038/s41423-020-0488-6 PMC7395159 32612154

[55] Poorebrahim M Sadeghi S Fakhr E Abazari MF Poortahmasebi V Kheirollahi A. Production of CAR T-cells by GMP-grade lentiviral vectors: latest advances and future prospects. Crit Rev Clin Lab Sci. (2019) 56:393–419. doi: 10.1080/10408363.2019.1633512 31314617

[56] Banker DD. Monoclonal antibodies. A review. In Indian J Med Sci. (2001) 55:651–4. doi: 10.2174/1574884712666170809124728 12024990

[57] Salles G Barrett M Foà R Maurer J O’Brien S Valente N. Rituximab in B-cell hematologic Malignancies: A review of 20 years of clinical experience. Adv Ther. (2017) 34:2232–73. doi: 10.1007/s12325-017-0612-x PMC5656728 28983798

[58] Marin-Acevedo JA Soyano AE Dholaria B Knutson KL Lou Y. Cancer immunotherapy beyond immune checkpoint inhibitors. J Hematol Oncol. (2018) 11:8. doi: 10.1186/s13045-017-0552-6 29329556 PMC5767051

[59] Shiravand Y Khodadadi F Kashani SMA Hosseini-Fard SR Hosseini S Sadeghirad H. Immune checkpoint inhibitors in cancer therapy. Curr Oncol (Toronto Ont.). (2022) 29:3044–60. doi: 10.3390/curroncol29050247 PMC9139602 35621637

[60] Fan J Shang D Han B Song J Chen H Yang JM. Adoptive cell transfer: is it a promising immunotherapy for colorectal cancer? Theranostics. (2018) 8:5784–800. doi: 10.7150/thno.29035 PMC6276301 30555581

[61] Grimm EA Mazumder A Zhang HZ Rosenberg SA. Lymphokine-activated killer cell phenomenon. Lysis of natural killer-resistant fresh solid tumor cells by interleukin 2-activated autologous human peripheral blood lymphocytes. J Exp Med. (1982) 155:1823–41. doi: 10.1084/jem.155.6.1823 PMC2186695 6176669

[62] Rosenberg SA. Immunotherapy of patients with advanced cancer using interleukin-2 alone or in combination with lymphokine activated killer cells. Important Adv Oncol. (1988) 19: 217–57. doi: 10.1186/s12916-021-02006-4 3042605

[63] Gorabi AM Hajighasemi S Sathyapalan T Sahebkar A. Cell transfer-based immunotherapies in cancer: A review. IUBMB Life. (2020) 72:790–800. doi: 10.1002/iub.2180 31633881

[64] Wang S Sun J Chen K Ma P Lei Q Xing S. Perspectives of tumor-infiltrating lymphocyte treatment in solid tumors. BMC Med. (2021) 19:140. doi: 10.1186/s12916-021-02006-4 34112147 PMC8194199

[65] Hughes-Parry HE Cross RS Jenkins MR. The evolving protein engineering in the design of chimeric antigen receptor T cells. Int J Mol Sci. (2019) 21. doi: 10.3390/ijms21010204 PMC6981602 31892219

[66] Chen YJ Abila B Mostafa Kamel Y. CAR-T: what is next? Cancers. (2023) 15. doi: 10.3390/cancers15030663 PMC9913679 36765623

[67] Labanieh L Majzner RG Mackall CL. Programming CAR-T cells to kill cancer. Nat Biomed Eng. (2018) 2:377–91. doi: 10.1038/s41551-018-0235-9 31011197

[68] Zhang C Liu J Zhong JF Zhang X. Engineering CAR-T cells. biomark Res. (2017) 5:22. doi: 10.1186/s40364-017-0102-y 28652918 PMC5482931

[69] Dotti G Gottschalk S Savoldo B Brenner MK. Design and development of therapies using chimeric antigen receptor-expressing T cells. Immunol Rev. (2014) 257:107–26. doi: 10.1111/imr.12131 PMC3874724 24329793

[70] Benmebarek MR Karches CH Cadilha BL Lesch S Endres S Kobold S. Killing mechanisms of chimeric antigen receptor (CAR) T cells. Int J Mol Sci. (2019) 20. doi: 10.3390/ijms20061283 PMC6470706 30875739

[71] Safarzadeh Kozani P Naseri A Mirarefin SMJ Salem F Nikbakht M Evazi Bakhshi S. Nanobody-based CAR-T cells for cancer immunotherapy. biomark Res. (2022) 10:24. doi: 10.1186/s40364-022-00371-7 35468841 PMC9036779

[72] Feins S Kong W Williams EF Milone MC Fraietta JA. An introduction to chimeric antigen receptor (CAR) T-cell immunotherapy for human cancer. Am J Hematol. (2019) 94:S3–9. doi: 10.1002/ajh.25418 30680780

[73] Stone JD Kranz DM. Role of T cell receptor affinity in the efficacy and specificity of adoptive T cell therapies. Front Immunol. (2013) 4:244. doi: 10.3389/fimmu.2013.00244 23970885 PMC3748443

[74] Gill S Porter DL. CAR-modified anti-CD19 T cells for the treatment of B-cell Malignancies: Rules of the road. In Expert Opin Biol Ther. (2014) 14:37–49). doi: 10.1517/14712598.2014.860442 24261468

[75] Li D Wang R Liang T Ren H Park C Tai CH. Camel nanobody-based B7-H3 CAR-T cells show high efficacy against large solid tumours. Nat Commun. (2023) 14. doi: 10.1038/s41467-023-41631-w PMC10517151 37739951

[76] Bao C Gao Q Li L-L Han L Zhang B Ding Y. The application of nanobody in CAR-T therapy. Biomolecules. (2021) 11. doi: 10.3390/biom11020238 PMC7914546 33567640

[77] Muyldermans S. Nanobodies: natural single-domain antibodies. Annu Rev Biochem. (2013) 82:775–97. doi: 10.1146/annurev-biochem-063011-092449 23495938

[78] Sun W Xie J Lin H Mi S Li Z Hua F. A combined strategy improves the solubility of aggregation-prone single-chain variable fragment antibodies. Protein Expression Purification. (2012) 83:21–9. doi: 10.1016/j.pep.2012.02.006 22387083

[79] Faitschuk E Nagy V Hombach AA Abken H. A dual chain chimeric antigen receptor (CAR) in the native antibody format for targeting immune cells towards cancer cells without the need of an scFv. Gene Ther. (2016) 23:718–26. doi: 10.1038/gt.2016.48 27356950

[80] Elahi R Khosh E Tahmasebi S Esmaeilzadeh A. Immune cell hacking: challenges and clinical approaches to create smarter generations of chimeric antigen receptor T cells. Front Immunol. (2018) 9:1717. doi: 10.3389/fimmu.2018.01717 30108584 PMC6080612

[81] Jayaraman J Mellody MP Hou AJ Desai RP Fung AW Pham AHT. CAR-T design: Elements and their synergistic function. EBioMedicine. (2020) 58:102931. doi: 10.1016/j.ebiom.2020.102931 32739874 PMC7393540

[82] Jonnalagadda M Mardiros A Urak R Wang X Hoffman LJ Bernanke A. Chimeric antigen receptors with mutated igG4 fc spacer avoid fc receptor binding and improve T cell persistence and antitumor efficacy. Mol Ther. (2015) 23:757. doi: 10.1038/mt.2014.208 25366031 PMC4395772

[83] Rafiq S Hackett CS Brentjens RJ. Engineering strategies to overcome the current roadblocks in CAR T cell therapy. Nat Rev Clin Oncol. (2020) 17:147–67. doi: 10.1038/s41571-019-0297-y PMC7223338 31848460

[84] Andrea AE Chiron A Mallah S Bessoles S Sarrabayrouse G Hacein-Bey-Abina S. Advances in CAR-T cell genetic engineering strategies to overcome hurdles in solid tumors treatment. Front Immunol. (2022) 13:830292. doi: 10.3389/fimmu.2022.830292 35211124 PMC8861853

[85] Esensten JH Helou YA Chopra G Weiss A Bluestone JA. CD28 costimulation: from mechanism to therapy. Immunity. (2016) 44:973–88. doi: 10.1016/j.immuni.2016.04.020 PMC4932896 27192564

[86] Zapata JM Perez-Chacon G Carr-Baena P Martinez-Forero I Azpilikueta A Otano I. CD137 (4-1BB) signalosome: complexity is a matter of TRAFs. Front Immunol. (2018) 9:2618. doi: 10.3389/fimmu.2018.02618 30524423 PMC6262405

[87] Nair R Westin J. CAR T-cells. Adv Exp Med Biol. (2020) 1244:215–33. doi: 10.1007/978-3-030-41008-7_10 32301017

[88] Cui X Liu R Duan L Cao D Zhang Q Zhang A. CAR-T therapy: Prospects in targeting cancer stem cells. J Cell Mol Med. (2021) 25:9891–904. doi: 10.1111/jcmm.16939 PMC8572776 34585512

[89] Chmielewski M Hombach AA Abken H. Of CARs and TRUCKs: chimeric antigen receptor (CAR) T cells engineered with an inducible cytokine to modulate the tumor stroma. Immunol Rev. (2014) 257:83–90. doi: 10.1111/imr.12125 24329791

[90] Tokarew N Ogonek J Endres S von Bergwelt-Baildon M Kobold S. Teaching an old dog new tricks: next-generation CAR T cells. Br J Cancer. (2019) 120:26–37. doi: 10.1038/s41416-018-0325-1 30413825 PMC6325111

[91] Zhang W Jordan KR Schulte B Purev E. Characterization of clinical grade CD19 chimeric antigen receptor T cells produced using automated CliniMACS Prodigy system. Drug Design Dev Ther. (2018) 12:3343–56. doi: 10.2147/DDDT.S175113 PMC6181073 30323566

[92] Barrett DM Singh N Porter DL Grupp SA June CH. Chimeric antigen receptor therapy for cancer. Annu Rev Med. (2014) 65:333–47. doi: 10.1146/annurev-med-060512-150254 PMC4120077 24274181

[93] Abou-El-Enein M Elsallab M Feldman SA Fesnak AD Heslop HE Marks P. Scalable manufacturing of CAR T cells for cancer immunotherapy. Blood Cancer Discovery. (2021) 2:408–22. doi: 10.1158/2643-3230.BCD-21-0084 PMC8462122 34568831

[94] Roddie C O’Reilly M Dias Alves Pinto J Vispute K Lowdell M. Manufacturing chimeric antigen receptor T cells: issues and challenges. Cytotherapy. (2019) 21:327–40. doi: 10.1016/j.jcyt.2018.11.009 30685216

[95] Wang X Rivière I. Clinical manufacturing of CAR T cells: foundation of a promising therapy. Mol Ther Oncolytics. (2016) 3:16015. doi: 10.1038/mto.2016.15 27347557 PMC4909095

[96] Amini L Silbert SK Maude SL Nastoupil LJ Ramos CA Brentjens RJ. Preparing for CAR T cell therapy: patient selection, bridging therapies and lymphodepletion. Nat Rev Clin Oncol. (2022) 19:342–55. doi: 10.1038/s41571-022-00607-3 35318469

[97] Rosenberg SA. Finding suitable targets is the major obstacle to cancer gene therapy. Cancer Gene Ther. (2014) 21:45–7. doi: 10.1038/cgt.2014.3 PMC6354240 24535159

[98] Aparicio C Belver M Enríquez L Espeso F Núñez L Sánchez A. Cell therapy for colorectal cancer: the promise of chimeric antigen receptor (CAR)-T cells. Int J Mol Sci. (2021) 22:11781. doi: 10.3390/ijms222111781 34769211 PMC8583883

[99] Zhang Y Li Y Cao W Wang F Xie X Li Y. Single-cell analysis of target antigens of CAR-T reveals a potential landscape of “On-target, off-tumor toxicity”. Front Immunol. (2021) 12:799206. doi: 10.3389/fimmu.2021.799206 34975912 PMC8716389

[100] Ukrainskaya VM Musatova OE Volkov DV Osipova DS Pershin DS Moysenovich AM. CAR-tropic extracellular vesicles carry tumor-associated antigens and modulate CAR T cell functionality. Sci Rep. (2023) 13:463. doi: 10.1038/s41598-023-27604-5 36627334 PMC9832131

[101] Arabi F Torabi-Rahvar M Shariati A Ahmadbeigi N Naderi M. Antigenic targets of CAR T Cell Therapy. A retrospective view on clinical trials. Exp Cell Res. (2018) 369:1–10. doi: 10.1016/j.yexcr.2018.05.009 29758187

[102] An W Kang J-S Oh S Tu A. MST1R as a potential new target antigen of chimeric antigen receptor T cells to treat solid tumors. Korean J Physiol Pharmacology : Off J Korean Physiol Soc Korean Soc Pharmacol. (2023) 27:241–56. doi: 10.4196/kjpp.2023.27.3.241 PMC10122994 37078298

[103] Tian M Cheuk AT Wei JS Abdelmaksoud A Chou H-C Milewski D. An optimized bicistronic chimeric antigen receptor against GPC2 or CD276 overcomes heterogeneous expression in neuroblastoma. J Clin Invest. (2022) 132. doi: 10.1172/JCI155621 PMC9374382 35852863

[104] Wang A Lv T Song Y. Tandem CAR-T cells targeting MUC1 and PSCA combined with anti-PD-1 antibody exhibit potent preclinical activity against non-small cell lung cancer. Cell Immunol. (2023) 391–392:104760. doi: 10.1016/j.cellimm.2023.104760 37660477

[105] Moretti A Ponzo M Nicolette CA Tcherepanova IY Biondi A Magnani CF. The past, present, and future of non-viral CAR T cells. Front Immunol. (2022) 13:867013. doi: 10.3389/fimmu.2022.867013 35757746 PMC9218214

[106] Milone MC O’Doherty U. Clinical use of lentiviral vectors. Leukemia. (2018) 32:1529–41. doi: 10.1038/s41375-018-0106-0 PMC6035154 29654266

[107] Gross G Waks T Eshhar Z. Expression of immunoglobulin-T-cell receptor chimeric molecules as functional receptors with antibody-type specificity. Proc Natl Acad Sci. (1989) 86:10024–8. doi: 10.1073/pnas.86.24.10024 PMC298636 2513569

[108] Zhang Z Miao L Ren Z Tang F Li Y. Gene-edited interleukin CAR-T cells therapy in the treatment of Malignancies: present and future. Front Immunol. (2021) 12:718686. doi: 10.3389/fimmu.2021.718686 34386015 PMC8353254

[109] Albelda SM. CAR T cell therapy for patients with solid tumours: key lessons to learn and unlearn. Nat Rev Clin Oncol. (2024) 21:47–66. doi: 10.1038/s41571-023-00832-4 37904019

[110] Barros LRC Couto SCF da Silva Santurio D Paixão EA Cardoso F da Silva VJ. Systematic review of available CAR-T cell trials around the world. Cancers. (2022) 14:2667. doi: 10.3390/cancers14112667 35681646 PMC9179563

[111] Patel U Abernathy J Savani BN Oluwole O Sengsayadeth S Dholaria B. CAR T cell therapy in solid tumors: A review of current clinical trials. EJHaem. (2022) 3:24–31. doi: 10.1002/jha2.356 35844304 PMC9175685

[112] Yan T Zhu L Chen J. Current advances and challenges in CAR T-Cell therapy for solid tumors: tumor-associated antigens and the tumor microenvironment. Exp Hematol Oncol. (2023) 12:14. doi: 10.1186/s40164-023-00373-7 36707873 PMC9883880

[113] Maude S Barrett DM. Current status of chimeric antigen receptor therapy for haematological Malignancies. Br J Haematology. (2016) 172:11–22. doi: 10.1111/bjh.13792 26560054

[114] Martino M Alati C Canale FA Musuraca G Martinelli G Cerchione C. A review of clinical outcomes of CAR T-cell therapies for B-acute lymphoblastic leukemia. Int J Mol Sci. (2021) 22. doi: 10.3390/ijms22042150 PMC7926700 33670075

[115] Todorovic Z Todorovic D Markovic V Ladjevac N Zdravkovic N Djurdjevic P. CAR T cell therapy for chronic lymphocytic leukemia: successes and shortcomings. Curr Oncol (Toronto Ont.). (2022) 29:3647–57. doi: 10.3390/curroncol29050293 PMC9139644 35621683

[116] Wei W Yang D Chen X Liang D Zou L Zhao X. Chimeric antigen receptor T-cell therapy for T-ALL and AML. Front Oncol. (2022) 12:967754. doi: 10.3389/fonc.2022.967754 36523990 PMC9745195

[117] Wang X Zhang Y Xue S. Recent progress in chimeric antigen receptor therapy for acute myeloid leukemia. Ann Hematol. (2024) 4: 1–15. doi: 10.1007/S00277-023-05601-Y/METRICS 38381173

[118] Arcangeli S Rotiroti MC Bardelli M Simonelli L Magnani CF Biondi A. Balance of anti-CD123 chimeric antigen receptor binding affinity and density for the targeting of acute myeloid leukemia. Mol Ther. (2017) 25:1933. doi: 10.1016/j.ymthe.2017.04.017 28479045 PMC5542631

[119] Ehninger A Kramer M Röllig C Thiede C Bornhäuser M Von Bonin M. Distribution and levels of cell surface expression of CD33 and CD123 in acute myeloid leukemia. Blood Cancer J. (2014) 4. doi: 10.1038/bcj.2014.39 PMC4080210 24927407

[120] Wang QS Wang Y Lv HY Han QW Fan H Guo B. Treatment of CD33-directed chimeric antigen receptor-modified T cells in one patient with relapsed and refractory acute myeloid leukemia. Mol Therapy : J Am Soc Gene Ther. (2015) 23:184–91. doi: 10.1038/mt.2014.164 PMC4426796 25174587

[121] Daver N Alotaibi AS Bücklein V Subklewe M. T-cell-based immunotherapy of acute myeloid leukemia: current concepts and future developments. Leukemia. (2021) 35:1843–63. doi: 10.1038/s41375-021-01253-x PMC8257483 33953290

[122] Liu F Cao Y Pinz K Ma Y Wada M Chen K. First-in-human CLL1-CD33 compound CAR T cell therapy induces complete remission in patients with refractory acute myeloid leukemia: update on phase 1 clinical trial. Blood. (2018) 132:901–1. doi: 10.1182/blood-2018-99-110579

[123] Abramson JS Lunning M Palomba ML. Chimeric antigen receptor T-cell therapies for aggressive B-cell lymphomas: current and future state of the art. Am Soc Clin Oncol Educ Book. Am Soc Clin Oncol Annu Meeting. (2019) 39:446–53. doi: 10.1200/EDBK_238693 31099671

[124] Ormhøj M Bedoya F Frigault MJ Maus MV. CARs in the lead against Multiple Myeloma. Curr Hematologic Malignancy Rep. (2017) 12:119. doi: 10.1007/s11899-017-0373-2 PMC5410390 28233151

[125] Martino M Canale FA Alati C Vincelli ID Moscato T Porto G. CART-cell therapy: recent advances and new evidence in multiple myeloma. Cancers. (2021) 13. doi: 10.3390/cancers13112639 PMC8197914 34072068

[126] Alcantara M Du Rusquec P Romano E. Current clinical evidence and potential solutions to increase benefit of CAR T-cell therapy for patients with solid tumors. OncoImmunology. (2020) 9. doi: 10.1080/2162402X.2020.1777064 PMC7466853 32934880

[127] Martinez M Moon EK. CAR T cells for solid tumors: new strategies for finding, infiltrating, and surviving in the tumor microenvironment. Front Immunol. (2019) 10:128. doi: 10.3389/fimmu.2019.00128 30804938 PMC6370640

[128] Ma S Li X Wang X Cheng L Li Z Zhang C. Current progress in CAR-T cell therapy for solid tumors. Int J Biol Sci. (2019) 15:2548–60. doi: 10.7150/ijbs.34213 PMC6854376 31754328

[129] Kieliszek AM Mobilio D Upreti D Bloemberg D Escudero L Kwiecien JM. Intratumoral delivery of chimeric antigen receptor T cells targeting CD133 effectively treats brain metastases. Clin Cancer Res. (2023) 185: OF1–OF10. doi: 10.1158/1078-0432.CCR-23-1735 37787999

[130] Alsajjan R Mason WP. Bispecific T-cell engagers and chimeric antigen receptor T-cell therapies in glioblastoma: an update. Curr Oncol (Toronto Ont.). (2023) 30:8501–49. doi: 10.3390/curroncol30090619 PMC10529026 37754534

[131] Baker DJ June CH. CAR T therapy extends its reach to autoimmune diseases. Cell. (2022) 185:4471–3. doi: 10.1016/j.cell.2022.10.026 PMC13041721 36423579

[132] Mackensen A Müller F Mougiakakos D Böltz S Wilhelm A Aigner M. Anti-CD19 CAR T cell therapy for refractory systemic lupus erythematosus. Nat Med. (2022) 28:2124–32. doi: 10.1038/s41591-022-02017-5 36109639

[133] Mackensen A Müller F Mougiakakos D Böltz S Wilhelm A Aigner M. Author Correction: Anti-CD19 CAR T cell therapy for refractory systemic lupus erythematosus. Nat Med. (2023) 29:2956. doi: 10.1038/s41591-022-02091-9 36329355

[134] Sakaguchi S Yamaguchi T Nomura T Ono M. Regulatory T cells and immune tolerance. Cell. (2008) 133:775–87. doi: 10.1016/j.cell.2008.05.009 18510923

[135] Wyss L Stadinski BD King CG Schallenberg S Mccarthy NI Lee JY. Affinity for self antigen selects Treg cells with distinct functional properties. Nat Immunol. (2016) 17:1093–101. doi: 10.1038/ni.3522 PMC4994872 27478940

[136] Requejo Cier CJ Valentini N Lamarche C. Unlocking the potential of Tregs: innovations in CAR technology. Front Mol Biosci. (2023) 10:1267762. doi: 10.3389/fmolb.2023.1267762 37900916 PMC10602912

[137] Shimabukuro-Vornhagen A Gödel P Subklewe M Stemmler HJ Schlößer HA Schlaak M. Cytokine release syndrome. J Immunotherapy Cancer. (2018) 6:56. doi: 10.1186/s40425-018-0343-9 PMC6003181 29907163

[138] Zhang Q Ping J Huang Z Zhang X Zhou J Wang G. CAR-T cell therapy in cancer: tribulations and road ahead. J Immunol Res. (2020) 2020:1924379. doi: 10.1155/2020/1924379 32411789 PMC7201836

[139] Qi X Li J Luo P. Glycyrrhizin for treatment of CRS caused by CAR T-cell therapy: A pharmacological perspective. Front Pharmacol. (2023) 14:1134174. doi: 10.3389/fphar.2023.1134174 36923358 PMC10009180

[140] Kotch C Barrett D Teachey DT. Tocilizumab for the treatment of chimeric antigen receptor T cell-induced cytokine release syndrome. Expert Rev Clin Immunol. (2019) 15:813–22. doi: 10.1080/1744666X.2019.1629904 PMC7936577 31219357

[141] Caimi PF Pacheco Sanchez G Sharma A Otegbeye F Ahmed N Rojas P. Prophylactic tocilizumab prior to anti-CD19 CAR-T cell therapy for non-hodgkin lymphoma. Front Immunol. (2021) 12:745320. doi: 10.3389/fimmu.2021.745320 34712233 PMC8546323

[142] Lakomy T Akhoundova D Nilius H Kronig M-N Novak U Daskalakis M. Early Use of Corticosteroids following CAR T-Cell Therapy Correlates with Reduced Risk of High-Grade CRS without Negative Impact on Neurotoxicity or Treatment Outcome. Biomolecules. (2023) 13:382. doi: 10.3390/biom13020382 36830750 PMC9953517

[143] Gazeau N Liang EC Wu QV Voutsinas JM Barba P Iacoboni G. Anakinra for refractory cytokine release syndrome or immune effector cell-Associated neurotoxicity syndrome after chimeric antigen receptor T cell therapy. Transplant Cell Ther. (2023) 29:430–7. doi: 10.1016/j.jtct.2023.04.001 PMC10330552 37031746

[144] Yu S Yi M Qin S Wu K. Next generation chimeric antigen receptor T cells: safety strategies to overcome toxicity. Mol Cancer. (2019) 18. doi: 10.1186/s12943-019-1057-4 PMC6701025 31429760

[145] Liang Q Monetti C Shutova MV Neely EJ Hacibekiroglu S Yang H. Linking a cell-division gene and a suicide gene to define and improve cell therapy safety. Nature. (2018) 563:701–4. doi: 10.1038/s41586-018-0733-7 30429614

[146] Zheng Y Nandakumar KS Cheng K. Optimization of CAR-T cell-based therapies using small-molecule-based safety switches. J Medicinal Chem. (2021) 64:9577–91. doi: 10.1021/acs.jmedchem.0c02054 34191515

[147] Xiong X Yu Y Jin X Xie D Sun R Lu W. Functional validation of the RQR8 suicide /marker gene in CD19 CAR-T cells and CLL1CAR-T cells. Ann Hematol. (2023) 102:1523–35. doi: 10.1007/s00277-023-05227-0 37086278

[148] Tiberghien P. Use of suicide genes in gene therapy. J Leukocyte Biol. (1994) 56:203–9. doi: 10.1002/jlb.56.2.203 8071596

[149] Sułkowski M Konieczny P Chlebanowska P Majka M. Introduction of exogenous HSV-TK suicide gene increases safety of keratinocyte-derived induced pluripotent stem cells by providing genetic “Emergency exit” Switch. Int J Mol Sci. (2018) 19:197. doi: 10.3390/ijms19010197 29315221 PMC5796146

[150] Rossignoli F Grisendi G Spano C Golinelli G Recchia A Rovesti G. Inducible Caspase9-mediated suicide gene for MSC-based cancer gene therapy. Cancer Gene Ther. (2019) 26:11–6. doi: 10.1038/s41417-018-0034-1 PMC6760542 29955091

[151] Li P Nijhawan D Budihardjo I Srinivasula SM Ahmad M Alnemri ES. Cytochrome c and dATP-Dependent Formation of Apaf-1/Caspase-9 Complex Initiates an Apoptotic Protease Cascade. Cell. (1997) 91:479–89. doi: 10.1016/S0092-8674(00)80434-1 9390557

[152] Gargett T Brown MP. The inducible caspase-9 suicide gene system as a “safety switch” to limit on-target, off-tumor toxicities of chimeric antigen receptor T cells. Front Pharmacol. (2014) 5:235. doi: 10.3389/fphar.2014.00235 25389405 PMC4211380

[153] Zhou X Dotti G Krance RA Martinez CA Naik S Kamble RT. Inducible caspase-9 suicide gene controls adverse effects from alloreplete T cells after haploidentical stem cell transplantation. Blood. (2015) 125:4103–13. doi: 10.1182/blood-2015-02-628354 PMC4481597 25977584

[154] Gumber D Wang LD. Improving CAR-T immunotherapy: Overcoming the challenges of T cell exhaustion. EBioMedicine. (2022) 77:103941. doi: 10.1016/j.ebiom.2022.103941 35301179 PMC8927848

[155] Mestermann K Giavridis T Weber J Rydzek J Frenz S Nerreter T. The tyrosine kinase inhibitor dasatinib acts as a pharmacologic on/off switch for CAR T cells. Sci Trans Med. (2019) 11. doi: 10.1126/scitranslmed.aau5907 PMC7523030 31270272

[156] Mishra A Maiti R Mohan P Gupta P. Antigen loss following CAR-T cell therapy: Mechanisms, implications, and potential solutions. Eur J Haematol. (2023) 9(38):1–16. doi: 10.1111/ejh.14101 37705357

[157] Zhao N Hu F Zhai Y Ye X Ruan Y Liu Z. Ocular toxicities in chimeric antigen receptor T-cell therapy: a real-world study leveraging FAERS database. Immunotherapy. (2023) 25:625–38. doi: 10.2217/imt-2023-0220 38126138

[158] Rejeski K Perez A Iacoboni G Blumenberg V Bücklein VL Völkl S. Severe hematotoxicity after CD19 CAR-T therapy is associated with suppressive immune dysregulation and limited CAR-T expansion. Sci Adv. (2023) 9. doi: 10.1126/sciadv.adg3919 PMC10516499 37738350

[159] Lee DW Santomasso BD Locke FL Ghobadi A Turtle CJ Brudno JN. ASTCT consensus grading for cytokine release syndrome and neurologic toxicity associated with immune effector cells. Biol Blood Marrow Transplant. (2019) 25:625–38. doi: 10.1016/j.bbmt.2018.12.758 PMC12180426 30592986

[160] Introna M Barbui AM Bambacioni F Casati C Gaipa G Borleri G. Genetic modification of human T cells with CD20: A strategy to purify and lyse transduced cells with anti-CD20 antibodies. Hum Gene Ther. (2000) 11:611–20. doi: 10.1089/10430340050015798 10724039

[161] Kao RL Truscott LC Chiou T-T Tsai W Wu AM De Oliveira SN. A cetuximab-mediated suicide system in chimeric antigen receptor–modified hematopoietic stem cells for cancer therapy. Hum Gene Ther. (2019) 30:413–28. doi: 10.1089/hum.2018.180 PMC6479239 30860401

[162] Xu N Yang X-F Xue S-L Tan J-W Li M-H Ye J. Ruxolitinib reduces severe CRS response by suspending CAR-T cell function instead of damaging CAR-T cells. Biochem Biophys Res Commun. (2022) 595:54–61. doi: 10.1016/j.bbrc.2022.01.070 35101664

[163] Ali Hosseini Rad SM Poudel A Tan GMY McLellan AD. Optimisation of Tet-On inducible systems for Sleeping Beauty-based chimeric antigen receptor (CAR) applications. Sci Rep. (2020) 10:13125. doi: 10.1038/s41598-020-70022-0 32753634 PMC7403325

[164] Tristán-Manzano M Maldonado-Pérez N Justicia-Lirio P Cortijo-Gutierréz M Tristán-Ramos P Blanco-Benítez C. Lentiviral vectors for inducible, transactivator-free advanced therapy medicinal products: Application to CAR-T cells. Mol Ther - Nucleic Acids. (2023) 32:322–39. doi: 10.1016/j.omtn.2023.03.018 PMC10141506 37125150

[165] Zhang R-Y Wei D Liu Z-K Yong Y-L Wei W Zhang Z-Y. Doxycycline inducible chimeric antigen receptor T cells targeting CD147 for hepatocellular carcinoma therapy. Front Cell Dev Biol. (2019) 7:233. doi: 10.3389/fcell.2019.00233 31681766 PMC6798074

[166] Yang L Yin J Wu J Qiao L Zhao EM Cai F. Engineering genetic devices for in *vivo* control of therapeutic T cell activity triggered by the dietary molecule resveratrol. Proc Natl Acad Sci. (2021) 118. doi: 10.1073/pnas.2106612118 PMC8403971 34404729

[167] Kotter B Engert F Krueger W Roy A Rawashdeh W Cordes N. Titratable pharmacological regulation of CAR T cells using zinc finger-based transcription factors. Cancers. (2021) 13:4741. doi: 10.3390/cancers13194741 34638227 PMC8507528

[168] Miller IC Zamat A Sun L-K Phuengkham H Harris AM Gamboa L. Enhanced intratumoural activity of CAR T cells engineered to produce immunomodulators under photothermal control. Nat Biomed Eng. (2021) 5:1348–59. doi: 10.1038/s41551-021-00781-2 PMC8791016 34385695

[169] Yin Q Tang J Zhu X. Next-generation sequencing technologies accelerate advances in T-cell therapy for cancer. Briefings Funct Genomics. (2019) 18:119–28. doi: 10.1093/bfgp/ely018 PMC6488971 29982317

[170] Palmirotta R Lovero D Cafforio P Felici C Mannavola F Pellè E. Liquid biopsy of cancer: a multimodal diagnostic tool in clinical oncology. Ther Adv Med Oncol. (2018) 10:175883591879463. doi: 10.1177/1758835918794630 PMC6116068 30181785

[171] Tan WCC Nerurkar SN Cai HY Ng HHM Wu D Wee YTF. Overview of multiplex immunohistochemistry/immunofluorescence techniques in the era of cancer immunotherapy. Cancer Commun. (2020) 40:135–53. doi: 10.1002/cac2.12023 PMC7170662 32301585

[172] Iyer A Hamers AAJ Pillai AB. CyTOF® for the masses. Front Immunol. (2022) 13:815828. doi: 10.3389/fimmu.2022.815828 35493491 PMC9047695

[173] Minott JA van Vloten JP Yates JGE Chan L Wood GA Viloria-Petit AM. Multiplex flow cytometry-based assay for quantifying tumor- and virus-associated antibodies induced by immunotherapies. Front Immunol. (2022) 13:1038340. doi: 10.3389/fimmu.2022.1038340 36466867 PMC9708883

[174] Celichowski P Turi M Charvátová S Radhakrishnan D Feizi N Chyra Z. Tuning CARs: recent advances in modulating chimeric antigen receptor (CAR) T cell activity for improved safety, efficacy, and flexibility. J Trans Med. (2023) 21:197. doi: 10.1186/s12967-023-04041-6 PMC10015723 36922828

[175] Depil S Duchateau P Grupp SA Mufti G Poirot L. ‘Off-the-shelf’ allogeneic CAR T cells: development and challenges. Nat Rev Drug Discovery. (2020) 19:185–99. doi: 10.1038/s41573-019-0051-2 31900462

[176] Morgan MA Büning H Sauer M Schambach A. Use of cell and genome modification technologies to generate improved “Off-the-shelf” CAR T and CAR NK cells. Front Immunol. (2020) 11:1965. doi: 10.3389/fimmu.2020.01965 32903482 PMC7438733

[177] Nianias A Themeli M. Induced pluripotent stem cell (iPSC)–derived lymphocytes for adoptive cell immunotherapy: recent advances and challenges. Curr Hematologic Malignancy Rep. (2019) 14:261. doi: 10.1007/s11899-019-00528-6 PMC6647376 31243643

[178] Torikai H Reik A Liu PQ Zhou Y Zhang L Maiti S. A foundation for universal T-cell based immunotherapy: T cells engineered to express a CD19-specific chimeric-antigen-receptor and eliminate expression of endogenous TCR. Blood. (2012) 119:5697–705. doi: 10.1182/blood-2012-01-405365 PMC3382929 22535661

[179] Moradi V Omidkhoda A Ahmadbeigi N. The paths and challenges of “off-the-shelf” CAR-T cell therapy: An overview of clinical trials. Biomedicine Pharmacotherapy. (2023) 169:115888. doi: 10.1016/j.biopha.2023.115888 37979380

[180] Benjamin R Graham C Yallop D Jozwik A Mirci-Danicar OC Lucchini G. Genome-edited, donor-derived allogeneic anti-CD19 chimeric antigen receptor T cells in paediatric and adult B-cell acute lymphoblastic leukaemia: results of two phase 1 studies. Lancet. (2020) 396:1885–94. doi: 10.1016/S0140-6736(20)32334-5 PMC11773457 33308471

[181] Liu D Zhao J Song Y. Engineering switchable and programmable universal CARs for CAR T therapy. J Hematol Oncol. (2019) 12. doi: 10.1186/s13045-019-0763-0 PMC6610960 31272471

[182] Hu D Yang R Wang G Li H Fan X Liang G. Emerging strategies to overcome current CAR-T therapy dilemmas - exosomes derived from CAR-T cells. Int J Nanomedicine. (2024) 19:2773–91. doi: 10.2147/IJN.S445101 PMC10959326 38525009

[183] Gross G Eshhar Z. Therapeutic potential of T cell chimeric antigen receptors (CARs) in cancer treatment: counteracting off-tumor toxicities for safe CAR T cell therapy. Annu Rev Pharmacol Toxicol. (2016) 56:59–83. doi: 10.1146/annurev-pharmtox-010814-124844 26738472

[184] Al-Haideri M Tondok SB Safa SH Maleki AH Rostami S Jalil AT. CAR-T cell combination therapy: the next revolution in cancer treatment. Cancer Cell Int. (2022) 22:365. doi: 10.1186/s12935-022-02778-6 36419058 PMC9685957

