# Supplementary material for: CAR-T lymphocyte-based cell therapies; mechanistic substantiation, applications and biosafety enhancement with suicide genes: new opportunities to melt side effects
Source: Front Immunol. 2024 Jul 18;15:1333150. doi: 10.3389/fimmu.2024.1333150 (PMC11291200; doi:10.3389/fimmu.2024.1333150)
Supplement: Supplementary file 1 [file DataSheet_1.pdf]

# *Supplementary Material*

## 1 Supplementary Figures and Tables

### 1.1 Supplementary Tables

**Table S1:** Tumor-associated antigens actively investigated in clinical trials

| Antigen | Neoplasia | Potential off-tumor targets |
|---|---|---|
| **BCMA** | MM | B lymphocytes |
| **CAIX** | RCC, solid tumors in hypoxia | Gastric mucosa, pancreaticobiliary epithelium, crypts of the small intestine |
| **CD123** | AML | Myeloid progenitors, dendritic cells, B cells, macrophages, megakaryocytes, mastcells, monocytes, endothelial cells |
| **CD138** | MM | Epithelium, plasma cells and B-precursors |
| **CD19** | LLA, LLC, LNH, LH, LLP | Normal B lymphocytes |
| **CD20** | LLC, LNH | |
| **CD22** | ALL, NHL | |
| **CD30** | LNH, LTC, LH | Latent CD8 T lymphocytes, activated Th2 and B lymphocytes |
| **Antigen** | Neoplasia | Potential off-tumor targets |
| **BCMA** | MM | B lymphocytes |
| **CAIX** | RCC, solid tumors in hypoxia | Gastric mucosa, pancreaticobiliary epithelium, crypts of the small intestine |
| **CD123** | AML | Myeloid progenitors, dendritic cells, B lymphocytes, macrophages, megakaryocytes, mastcells, monocytes, endothelial cells |
| **CD138** | MM | Epithelium, plasma cells and B-precursors |
| **CD19** | LLA, LLC, LNH, LH, LLP | Normal B lymphocytes |
| **CS1** | MM | Plasma cell Natural killer,CD8 T-Lymphocytes, dendritic cells, activated monocytes |
| **CSPG4** | Melanoma, TNBC, GBM, mesothelioma, head and neck cancer, osteosarcoma | Basal cells of the epidermis, activated pericytesEndothelial cells |
| **EGFR** | Solid tumors | Tissues of mesenchymal origin, neuronal and epithelial |
| **EGFRvIII** | Cerebral/CNS, gliomas, GBM | None |
| **EphA2** | Glioma, breast, colon, ovarian cancer, prostate and pancreas | Endothelium |
| **ErbB2** | Brain cancer/CNS, GBM, glioma, head and neck, solid tumors | Gastrointestinal, reproductive, respiratory and urinary tract epithelia, skin, breast, haematopoietic cells |
| **FAP** | Mesothelioma | Fibroblasts in chronic inflammation |
| **FR-$\alpha$** | Ovarian cancer | Apical surface in kidney epithelia, Lung, Thyroid, Mamas |
| **GD2** | NB, sarcomas, solid tumors | Skin, neurons |
| **IgK** | LLC, LNH, MM | Normal B lymphocytes |
| **IL-11R$\alpha$** | Colon, breast, stomach, stomach, prostate and osteosarcoma cancers | Stromal tissue of gastrointestinal tract, endothelial cells, liver, glandular and surface epithelia |

| | | |
|---|---|---|
| **L1-CAM** | NB | Astrocytes Brain, head and neck tissue |
| **Lewis** | LMA, MM | CNS, SN sympatheticganglia Adrenalmedulla |
| **Mesothelin** | Mesothelioma ovarian and pancreaticcancer | Myeloid progenitor cells early |
| **MUC1** | Lung, breast, colon and ovarian cancer, kidney, stomach, prostate, head and neck | Pleural, pericardial and pericardialsurfaces peritoneal mesothelial |
| **NKG2D-L** | LMA, MM | Apical surface of most of the glandular epithelia |
| **PSCA** | Prostate, bladder and pancreatic cancer | Gastrointestinal epithelium, fibroblasts, endothelial cells |
| **PSMA** | Prostate cancer | Prostate |
| **ROR-1** | LLC, LNH | Apical surface of prostate andintestinal epithelia, proximal tubule cells |
| **VEGFR-2** | Solid tumors | Vascular and lymphatic endothelium |

Abbreviations: ALL, acute lymphoblastic leukaemia; AML, acute myeloid leukaemia; BCMA, B-cell maturation antigen; CAIX, carbonic anhydrase IX; CEA, carcinoembryonic antigen; CLL, chronic lymphocytic leukaemia; CNS, central nervous system; CSPG4, chondroitin sulphate proteoglycan 4; DC, dendritic cell; EGFR, epidermal growth factor receptor; EGFRvIII, EGFR variant III; EphA2, erythropoietin A2-producing hepatocellular carcinoma; FAP, fibroblast acti-vation protein; FR-α, folate receptor α; GBM, glioblastoma multiforme; HL, Hodgkins lymphoma; Ig, immunoglobulin; L1-CAM, L1 cell adhesion molecule; MM, multiple myeloma; NB, neuro-blastoma; NHL, non-Hodgkins lymphoma; PBMC, peripheral blood mononuclear cells; PLL prolymphocytic leukaemia; PSCA, prostate stem cell antigen; RCC, renal cell carcinoma; TCL, T-cell leukaemia/lymphoma; TNBC, triple negative breast cancer; VEGFR-2, vascular endothelial growth factor-2.Table adapted from (Dotti et al., 2014; Gross and Eshhar, 2016; Nair and Westin, 2020).

**Table S2:** FDA-approved CAR-T therapies until 2023

| Name | Trade name | Intracellular domain | Year of approval | Antigen | Disease | Clinical benefit |
|---|---|---|---|---|---|---|
| Tisagenlecleucel | Kymriah | CD3ζ 4-1BB | 2017 | CD19 | LLA-B | TR: 81%. \| CR: 60%. |
| | | | 2018 | | LDBG | ORR: 52%; \| CR: 40%; ORR: 52%; CR: 40%. |
| Axicabtageneciloleucel | Yescarta | CD3ζ CD28 | 2017 | CD19 | LDBG | ORR: 82%, \| CR:54%. |
| | | | 2021 | | LF | ORR: 91% : 91% \| CR: 60% |
| Brexucabtagene autoleucel | Tecartus | CD3ζ CD28 | 2020 | CD19 | LCM | ORR: 93%, \| CR: 67%. |
| Lisocabtagene maraleucel | Breyanzi | CD3ζ 4-1BB | 2021 | CD19 | LDBG | ORR: 74% CR:52% CR:52% ORR:74% |
| Idecabtagenevicleucel | Abecma | CD3ζ 4-1BB | 2021 | BCMA | MM | ORR:82%, \| CR: 39%. |
| Ciltacabtagene autoleucel | Carvykti | CD3ζ 4-1BB | 2022 | BCMA | MM | ORR: 95% \| CR:67% CR:67 |

B-ALL: acute lymphoblastic leukaemia; DLBCL: diffuse large B-cell lymphoma; MM: multiple myeloma; FL: follicular lymphoma; MCL: mantle cell lymphoma; CR: complete response; ORR: objective response ratio; ORR: objective response rate; RR: response rate All therapies are also EMA-approved except Citacabtagene autoleukel. Source adapted from: (Berdeja et al., 2021; Pan et al., 2022).

**Table S3:** CAR-T target antigens for the treatment of B-ALL together with the CAR structure target antigen.

| Target antigen | Structure |
|---|---|
| **CD19** | CD3ζ y CD28 o CD3ζ y 4-1BB |
| **CD22** | CD3ζ y CD28 |

**Table S4:** Molecules that participate in the antitumoral response and their respective functions

| Molecule | Role in the anti-tumor immune response |
|---|---|
| **IL-10** | It inhibits the immune response and limits excessive inflammation. |
| **IL-4** | Promotes Th2-type immune response and antibody production. |
| **CTLA-4** | It acts as a brake on the immune response by negatively regulating the activation of T cells. |
| **IL-13** | May have both promoting and inhibitory effects on the anti-tumor immune system. Mayactivate and recruit immune system cells and enhance antigen presentation tumors. |
| **PD-L1** | It acts as an inhibitory ligand for the PD-1 receptor on T-cells, regulating the immune response and preventing excessive responses. |
| **IDO** | It suppresses the immune response by depleting tryptophan and generating metabolites that inhibit T-cell proliferation. |
| **FasL** | It induces apoptosis (programmed cell death) in target cells, contributing to the elimination of damaged or infected cells. |
| **IL-2** | Stimulates the proliferation and differentiation of T cells, promoting their survival and activation. |
| **TNF-$\alpha$** | It is a pro-inflammatory cytokine involved in the anti-tumor immune response, promoting inflammation and destruction of tumor cells. |
| **IFN$\gamma$** | Stimulates cytotoxic activity of T cells and NK cells against tumor cells, as well as antigen presentation and the production of immunomodulatory molecules. |
| **IL-1** | Activates inflammatory responses and promotes T-cell and macrophage activation. |
| **IL-12** | Stimulates the production of interferon gamma (IFN$\gamma$) by T and NK cells, enhancing the anti-tumor immune response. |
| **CCL5** | Attraction and activation of immune system cells, such as T cells and NK cells, for target them to the tumor site and promote an anti-tumor immune response. |

## 1.2 Supplementary Figures

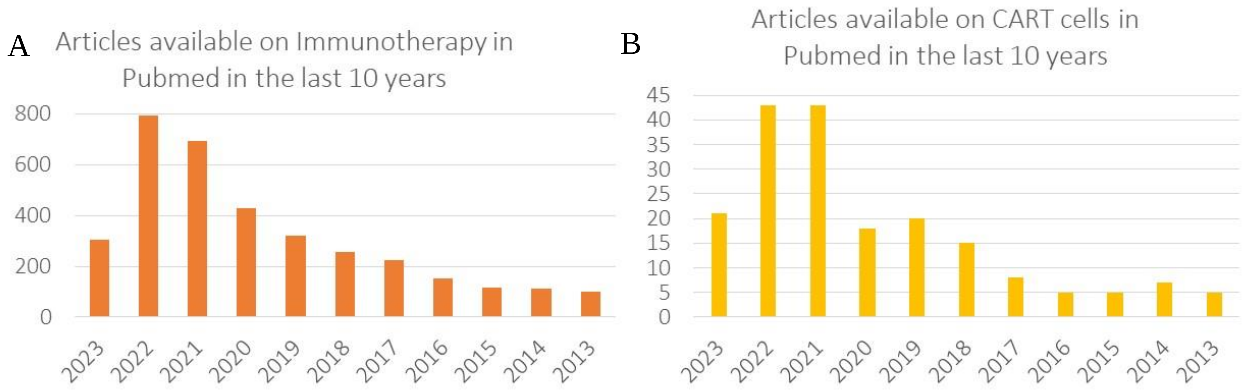


Figure S1: Representative graph of the articles available in the Pubmed database over the last 10 years. (A) Shows the number of articles on immunotherapy. (B) Shows the number of articles on CAR-T cells.
